# Supplementary material for: Understanding COVID-19 Vaccine Effectiveness against Death Using a Novel Measure: COVID Excess Mortality Percentage
Source: Vaccines (Basel). 2023 Feb 7;11(2):379. doi: 10.3390/vaccines11020379 (PMC9959409; doi:10.3390/vaccines11020379)

Supplementary Materials

Overview of Supplementary Materials

This supplementary material provides dataset and methods details, as well as additional results and robustness checks, for Atanasov et. al, COVID-19 Vaccine Effectiveness Against Death: Evidence from Linked Mortality and Vaccination Records, *Vaccines* (2023).

Contents

Additional Literature Discussion 2
Details on Data Sources, Data Linkage, and Match Accuracy 3
Identifying COVID-19 Decedents from Cause of Death Fields 4
Reporting Accuracy for COVID-19 Deaths and Excess Non-COVID-19 Natural Mortality 5
Virus Variants and Periods of Dominance 6
Measuring Population by Month and Number of Vaccine Doses 7
Time Period Studied, Variable Definitions. and Other Methods Details 7
Time Periods and Booster Rollout 9
Details on Younger, Fully Vaccinated COVID-19 Decedents 9
Vaccination Counts and Rates 9
Selection Effects for One-Dose Recipients 9
Confidence Intervals for Text Table 2 10
Additional Results for Text Table 2 10
Extended Multivariate Logit Results 10
Mortality-Weighted versus Population-Weighted Results 10
Defining the Immune-Compromised 10
J&J Vaccinees 11
Lag Period Between Vaccine Administration and Effectiveness Against Death 11
Results by Gender 12
Results for White versus Minority Decedents 12
Confidence Intervals for Text Table 3 12
RMRs Measured Using COVID-19 Mortality Rates Rather than CEMP 12
Compositional Effects for Full Sample Estimates 13
Evidence for Time-Varying Selection Effects and a Possibly Culling Effect 13
Additional References for supplementary materials (not cited in the text) 15
Table S1. Comparison of COVID-19 deaths per text analysis, to ICD-10 codes 16
Table S2. Summary Statistics on Vaccination Status 17
Table S3. Non-Covid Natural Mortality Rate: 1-, 2-, and 3-Dose Recipients 18
Table S4. Point Estimates and Confidence Intervals for RMR Estimates, Including One-Dose Vaccinees 19
Table S5. Vaccine Effectiveness (VE) by Age Group and Time Period: Additional Details 20
Table S6. Multivariate Logit Results for One-Dose Vaccinees and Finer Age Groups 21
Table S7. Population-Weighted CEMP and RMR by Time Period 22
Table S8. CEMP and RMR by Time Period, Including Immune-Compromised 23
Table S9. CEMP and RMR by Time Period, Excluding Immune-compromised and Decedents with Cancer 24
Table S10. CEMP and RMR by Time Period, Excluding J&J Vaccinees 25
Table S11. RMR by Time Period Using 14-day Minimum from Vaccination to Death 26

Table S12. CEMP and RMR by Time Period and Gender 27
Table S13. CEMP and RMR by Time Period and White vs. Minority 29
Table S14. Confidence Intervals for Non-Covid-NMR Estimates in Text Table 1...................... 31
Table S15. RMR Estimates Based on COVID-19 Mortality Rate.. 32
Table S16. Vaccinee counts by age and month 33
Figure S1. Sample selection flowchart 34
Figure S2. Correlation for Wisconsin and Milwaukee between Natural Mortality in 2019 and COVID-19 Mortality in 2020. 35
Figure S3. Vaccination Rates for Adults by Age Group in Milwaukee County.......................... 36
Figure S4. Actual versus Predicted Non-COVID Natural Mortality in Wisconsin During Pandemic Period, with Confidence Intervals.. 37
Figure S5. Actual versus Predicted Non-COVID Natural Mortality in Wisconsin During Pandemic Period, By Age Range... 38
Figure S6. COVID-19 Deaths Counts: Text Fields vs. ICD-10 Codes 39
Figure S7. Natural Deaths in 2019 vs. Non-COVID Natural Deaths in 2020............................. 40
Figure S8. Time-Varying Non-Covid-NMR Rates and Ratios... 41

Additional Literature Discussion

There are many studies of vaccine effectiveness, some of which include death as a studied outcome, a number of factors, taken together, sharply limit the universe of studies that we viewed as sufficiently comparable to our own to be worth citing. We applied the following exclusion criteria: First, the US used Pfizer and Moderna almost exclusively, with a bit of J&J (see counts by vaccine type for our sample in Table S2). In contrast, some European countries (in which we include the UK) used Astra-Zeneca extensively. (The US made limited use of the J&J single-dose vaccine, which most European countries did not use.) We therefore cite only studies that provide either vaccine-specific results, or combined results for the two mRNA vaccines (Pfizer and Moderna). Second, many studies cover limited time periods, before substantial waning had occurred. A number of studies of primary vaccination end in early to mid-2021, before substantial waning had occurred. We cite only studies that include all or most of 2021. Similarly, some booster studies cover very short time periods. Third, some studies cover limited populations (e.g., persons in care homes; healthcare workers; U.S. veterans; college students, etc.). We cited one only study of U.S. veterans which we viewed as well-done, other than the limited sample. Fourth, some studies cover only persons tested for COVID, persons already infected, or persons already hospitalized. Results from these studies cannot be compared to a population-based study such as ours because one important effect of vaccination was to reduce the risk of infection or hospitalization. Fifth, some studies rely on a combined outcome of hospitalization or death. These do not provide good comparisons for our study, which is only of mortality. Sixth, in the first half of 2021, when vaccines first became available, some countries (UK, Canada) stretched the manufacturer-recommended time gap between first and second doses well beyond the four weeks recommended by Moderna or the three weeks recommended by Pfizer, with the goal of providing first doses sooner, at the cost of delaying second doses. We excluded these studies because timing variation could affect two-dose effectiveness. Seventh, some studies do not include data on time since vaccination. Given the strong evidence of waning that we and other studies find, this is a crucial omission. Eighth, given the existence of a number of large-sample, country-level studies, studying populations in the millions, we excluded studies that used similar methods but had much

smaller samples. Finally, we excluded studies from Hong Kong, Japan, South Korea, Australia, New Zealand, and other countries which suppressed COVID infections to near zero prior to the emergence of the Omicron variant, because their experience may not be comparable to the rest of the world.

In addition to the studies noted in the text, we found a small study of Los Angeles County for May-July 2021, but this study had only 24 deaths among vaccinated persons and does not report RMR against death (Griffin et al., 2022).

As noted in the text, we found one study, of veterans during the Delta period, that found two-dose RMRs similar to those we report, for veterans seen at the VA, during 3Q-2021 (Delta period) (Cohn et al., 2022). This study controlled for age, sex, race, ethnicity, and comorbidities (Charlson comorbidity score, overweight, type II diabetes, chronic obstructive pulmonary disease, bronchitis, acute respiratory failure, and chronic lung disease). The study does not report results without these controls. These controls should reduce selection bias, and may on the whole, provide an adjustment for selection effects comparable to our control for natural non-COVID deaths.

The other study we found which controls for health is Xu et al. (Sweden), which also found substantial waning against death. However, this study censors persons who received a booster dose. This can introduce selection bias, with regard to which people receive a booster dose, despite the study's controls for health and other factors. Selection bias, and likely explains why this study reports lowest two-dose VE (highest RMR) 38- 45 weeks after second dose, with VE then oddly rising after that.

Our effort to control for background mortality risk is similar in spirit to a UK study of how VE varies based on body-mass-index (BMI) (Piernas et al., 2022).

Details on Data Sources, Data Linkage, and Match Accuracy

We obtain individual, de-identified mortality records for Milwaukee County for 2017 through June 2022 from the Wisconsin Department of Vital Statistics, and individual, de-identified COVID-19 vaccination records from the Wisconsin Immunization Registry for January 2021 through June 2022. The two sets of records include age in years, gender, 5-digit residence zip code, and linking "tokens" based on last name, first name, gender, and exact date of birth. In a small number of cases where first letter of first name was available but full first name was missing in one or both datasets, we accepted a match on last name, first initial of first name, gender, and date of birth.

Out of an estimated adult population of 722,000, of which 542,000 (75%) have vaccination records (Table S1), this match resulted in 1 apparent mismatch; a person who died of natural causes during our sample period, but was reported to have been vaccinated after death (although a correct match but an incorrect vaccination date is also possible).

For 955 people with vaccination records (0.18% of vaccinated persons), gender is reported as unknown in the vaccination records. The matching algorithm we use, from Datavant, treats missing gender as equivalent to male. Thus, if gender is male, the algorithm will match these people successfully to mortality records (which have complete data on gender), but if gender is female, no match will be found. Overall, we have 9,652 female deaths in our sample from natural causes (933 from COVID-19 and 8,719 from other natural causes). With an 0.18% missingness

rate (assuming missingness is similar for men and women), we will fail to match approximately 2 female COVID-19 deaths and 16 other natural deaths to vaccination records due to this issue. We have no reason to believe that these mismatches will lead to bias in CEMP values.

We define natural deaths as those with natural, pending, or undetermined manner of death; the vast majority (98.7%) have manner of death = natural. We exclude the remaining manner-of-death categories (accident, homicide, and suicide), which constitute 14.4% of all deaths.

Identifying COVID-19 Decedents from Cause of Death Fields

The death certificates that we rely on include text fields for primary cause of death, other conditions in the causal chain, and other significant conditions. We conduct text analysis of these fields to determine which deaths are likely to be caused by COVID-19. The small table below compares the COVID-19 counts we determine using text analysis to those from the ICD-10 codes included in death records, which are generated by personnel at the National Center for Health Statistics (NCHS) based on the text fields. Our text analysis algorithm was developed and substantially completed before we had access to the ICD-10 codes. Our goal was to develop a reasonable method for estimating COVID-19 deaths, based on the text fields in death certificates. The principal decisionmakers for this approach were Drs. John Meurer and Jeff Whittle.

Using ICD-10 codes produces many false negatives (deaths that we code as caused by COVID-19, but do not have COVID-19 coded as the primary cause of death, using ICD-10 code U07.1) and a smaller but still meaningful number of false positives (deaths that we code as not caused by COVID-19, but having U07.1 listed as the primary cause of death in the ICD-10 codes).

Most of the false negatives and false positives that would result from use of ICD-10 codes are not close cases. The NCHS coding is simply wrong, based on the text fields. Most of the false negatives involve COVID-19 deaths which were coded as B99 (other and unspecified infectious diseases). This coding error is largely limited to Milwaukee County; use of B99 in the rest of Wisconsin is rare. We speculate that the coder at NCHS responsible for Milwaukee death certificates did not realize there was a specific code for COVID-19 death. The false positives (deaths recorded with U07.1 as primary cause of death, that we coded as not due to COVID-19) typically lacked meaningful support from the text fields for COVID-19 as a cause of death.

Reliance on the text fields reduces classification error, in which CVOID deaths are misclassified as non-COVID or vice-versa, and thus improves the accuracy with which the CEMP measure controls for population health, as a predictor of COVID-19 mortality risk that is independent of vaccination status.

The detailed coding we used to identify deaths as due to or probably due to COVID-19 is available from the authors on request. In brief, we counted as COVID-19 deaths those for which:

(i) COVID-19 or variants (such as SARS-2, SARS-COV-2, coronavirus) was listed as the principal cause of death; or

(ii) COVID-19 or variants were listed in the causal chain and the principal cause of death was likely to be caused by COVID-19 (for example, pneumonia, respiratory failure, hypoxia); or

(iii) COVID-19 or variants were listed in the causal chain and the principal cause of death was a known potential outcome of COVID-19 infection (for example, heart attack, stroke,

sepsis) or involved underlying disease that was plausibly exacerbated by COVID-19 infection (for example, heart failure, COPD, dementia); or

(iv) COVID-19 was included in the "other significant conditions" field, but not in the causal chain fields, and (a) the principal cause of death was likely to be caused by COVID (group (ii) above); and (b) there was no entry in the causal chain fields indicating another likely cause of death.

We view reliability as high for categories (i) and (ii), and moderate for categories (iii) and (iv). The false positives (COVID-19 as principal cause of death based on ICD-10 codes, but not on our text-based coding) involve deaths for which the text fields do not fit in any of these four categories.

Reporting Accuracy for COVID-19 Deaths and Excess Non-COVID-19 Natural Mortality

We discuss in this section evidence on the accuracy of the text-based measure of COVID-19 deaths, and compare this measure to counts based on ICD-10 codes from NCHS. To the extent that our counts of COVID-19 deaths, although higher than NCHS counts, are still undercounts, this should show up in measures of excess non-COVID natural deaths, defined as non-COVID-19 natural deaths, minus predicted levels based on extrapolation of mortality trends from the pre-pandemic period. Undercounting of COVID-19 deaths would lead to excess deaths generally being positive during the pandemic periods, and spiking during the periods when measured COVID-19 mortality spikes. In text Figure 2, we show monthly measured COVID-19 deaths, measured non-COVID-19 natural deaths, and a prediction line based on extrapolation from the same calendar months (to allow for seasonality in natural deaths) from the pre-COVID period of 2017-2019. We report results for the full state of Wisconsin, rather than only Milwaukee County, to take advantage of the larger sample size available for the full states, which reduces the noise of monthly fluctuations in actual-minus-predicted non-COVID-19 natural deaths.

In this figure, there is no evidence for a substantial number of excess non-COVID-19 natural deaths. The predicted line is above the actual line roughly as often as below it. Moreover, there is no tendency for the actual-minus-predicted gap to be large during the two periods of high COVID-19 mortality, in late 2020 and late 2021-early 2022.

Another possibility, on which this figure also provides evidence, is that prior COVID-19 infection will lead to higher future deaths from natural causes, not directly linkable to the prior infection. Higher post-infection mortality would predict positive actual-minus-predicted non-COVID-19 natural mortality generally, not limited to periods of high COVID-19 mortality. There is evidence from other research of excess cardiovascular risk for a limited period of time following infection.[1] However, the extents of excess deaths is not known. Any excess deaths appear, from our Wisconsin data, not to be at levels apparent from the figure.

In Figure S4, we reproduce the measured and predicted counts of natural non-COVID deaths from text Figure 2, limited to the pandemic period, and include the 95% confidence interval (CI) around the predicted count. The measured count is never above the top of the CI for the predicted count.

The measure count falls below the bottom of the CI following the two large COVID death spiked, in late-2020 and late-2021 to early-2022. It is known that recent COVID-19 infection leads

[1] Rezel-Potts et. al (2022).

to somewhat higher risk for some causes of death in the next several months.[2] The comparison of actual to predicted non-COVID natural deaths in Figure S3 suggests that, if COVID-19 deaths are measured based on text fields in death certificates, a more important effect may be for the spikes in COVID-19 infection and deaths to have accelerated death for some people who would have died in the near future anyway.

In Figure S5, we provide graphs similar to text Figure 2 and Figure S3, but separate the sample into persons aged 18-59, 60-79, and 80+. Within each age range, there is again no evidence of substantial, excess natural non-COVID deaths, either during the peak periods for COVID deaths or soon after that.

Note from Figure S4 that natural non-Covid deaths are below predicted in 1H-2021, which both follows the late-2021 spike in COVID deaths, and is a period with high vaccination rates. During this period, natural non-COVID deaths are below those predicted by extrapolating from the pre-Covid period. The charts by age range show that the shortfall in natural non-Covid deaths is seen to some extent for ages 60-79, but is concentrated in ages 80+. A similar shortfall is observed for ages 80+ in 1H-22, following the COVID death spike in late 2021 and early 2022. This suggests the following tentative hypothesis, which would need to be tested using data from other states: To some extent, the COVID pandemic accelerated the deaths of already old, frail people, who had limited life expectancy but for COVID. These deaths contributed to the COVID spikes, but can also explain why natural, non-Covid deaths were below predicted levels for a period of time following each COVID spike.

In Figure S6, we provide further evidence on the choice between text-based counts of COVID-19 deaths and counts based on ICD-10 codes from NCHS. This figure shows COVID-19 deaths by month (measured using text fields) and the difference between the two counts, for Wisconsin (Panel A) and Indiana (Panel B). The text-based measure consistently counts more deaths, with the difference between the two measures spiking at the same times that COVID deaths spike. This provides strong evidence that the text-based measure performs better in capturing COVID deaths.

In Figure S7, we report evidence for Indiana (Panel A) and Milwaukee County (Panel B) on the correlation between natural deaths in 2019 (pre-COVID period) and measured non-COVID natural deaths in 2020, using the text-based measure, for groups defined by age (18-39, 40-49, 50-59, 60-69, 70-79, 80-89, 90+)*gender*race/ethnicity*zip-SES quintile. In Panel B, we study Milwaukee, rather than all of Wisconsin, due to limitations in the data available to us for the pre-COVID period. The Pearson correlation coefficients are 0.999 (Indiana) and 0.997 (Milwaukee County). These high correlations provide further evidence that the text-based measure does a good job of capturing COVID-19 deaths.

Virus Variants and Periods of Dominance

Accounting for the two-week lag we impose between dose administration and assumed effectiveness, the Alpha strain was dominant in the U.S. starting the week of March 14, 2021; the Delta variant was dominant beginning the week of July 4, 2021, and the Omicron variant has been

[2] Xie et. al (2022).

dominant starting the week of Jan. 2, 2022.[3] These periods correspond reasonably closely to the calendar quarters we use in text.

Measuring Population by Month and Number of Vaccine Doses

We construct a synthetic Milwaukee population by age-group and month as follows. We begin with American Community Survey (ACS) population estimates by age for 2020, which we assume apply to January 2020. The available age ranges in ACS are: 0-4; 5-9; 10-14; 15-17; 18-19; 20; 21; 22-24; 25-29; 30-34; 35-39; 40-44; 45-49; 50-54; 55-59; 60-61; 62-64; 65-66; 67-69; 70-74; 75-79; 80-84; and 85+. ACS provides only a single count for persons age 85+. Within each ACS age group we use the annual survival probabilities from NCHS by age and gender for 2018 (most recent year with available data) to divide the ACS population for that age group (in this example, ages 75-79) into an estimated number of persons age 75, 76, 77, 78, and 79. For the age 85+ group, we assume a maximum age of 100. Within each year of age, we assume birth month is random, so that approximately 1/12 of the population will be born in January, approximately 1/12 in February, and so on. We then roll forward a month at a time during the sample period. Thus, going from January to February 2020, everyone gets one month older and some (those assigned a January birth month) will be one year older. This gives us a starting synthetic population (before allowing for death) by year-month of age, for each calendar month.[4]

We next adjust this initial synthetic population for deaths. We ignore in- and out-migration, for which we lack data. From our mortality data, we have age at death in years; we assign month of birth randomly and compute age at death in months. In each calendar month we subtract the decedents from the synthetic population, to obtain a synthetic alive population by year-month of age for each calendar month. In a very small number of instances, for very advanced ages, this process results in a negative population count in an (year-month of age)*calendar month cell; we convert negative values to zero.

Table S16 provides estimated population counts by month and number of vaccine doses.

Time Period Studied, Variable Definitions. and Other Methods Details

COVID-19 vaccines because available in the U.S. in early 2021, but initially with limited supply, generally reserved the elderly, other persons at high risk for severe COVID-19, to healthcare workers, and other persons in high-exposure occupations. Availability expanded greatly in April 2021 and vaccines became available to all who wanted them by May 2021. We study mortality beginning April 1, 2021. Given the roughly one-month minimum period between first and second doses for both vaccines (3 weeks for Pfizer, 4 weeks for Moderna), and the minimum 30-day period we impose from most recent dose to death in order to treat a death as post-vaccination, it was not feasible to start the sample period much earlier than this.

---

[3] Source: https://www.gisaid.org/; see also https://covid.cdc.gov/covid-data-tracker/?utm_source=STAT+Newsletters&utm_campaign=059492f101-MR_COPY_01&utm_medium=email&utm_term=0_8cab1d7961-059492f101-153972538#variant-proportions

[4] We do not increase years of age to greater than 100 during this process.

CEMP, VE, and RMR

We define the COVID-19 Excess Mortality Percentage (CEMP), by number of doses *v*, and vaccine type, indexed by *i* (*i* = Pfizer, Moderna, or J&J), as the percentage increase in natural deaths due to COVID-19:

$$CEMP_{vi} = 100 \times \frac{COVID{-}19\ deaths_{vi}}{(Natural\ deaths - COVID19\ deaths)_{vi}} \quad (1)$$

We define VE and relative mortality risk after vaccination (RMR) by vaccine type, number of doses, and time period *t* as:

$$VE_{ivt} = \frac{(CEMP_{unvax,t} - CEMP_{vax,ivt})}{CEMP_{unvax,t}} \quad ; \quad RMR_{ivt} = 1 - VE_{ivt} = \frac{CEMP_{vax,ivt}}{CEMP_{unvax,t}} \quad (2)$$

These ratios will be undefined if the denominator is zero, but we did not encounter that issue for our sample.

We study one-dose and two-dose vaccinees in each time period, and three-dose vaccinees for the periods when a booster dose was available: fourth quarter of 2021 (“4Q-2021”) and the Omicron period (1H-2022). By using non-COVID natural deaths in the CEMP denominator, we treat the non-COVID natural mortality rate as a proxy for the overall health of a given group, which can control for selection effects in vaccination patterns, including who gets vaccinated, when, with which vaccine, and with how many doses. Because the sample is decedents, these estimates are, in effect, mortality-weighted; thus results for a broad age group give primary weight to older persons within that group.

CEMP represents the odds, for a population of decedents, of dying from COVID-19 versus dying other natural causes.

$$\frac{\sum COV_i^{mort} = 1}{\sum COV_i^{mort} = 0}$$

Here COV_i^{mort} equals 1 for COVID-19 decedents, 0 for decedents from other natural causes.

We define the RMR of one group *I* relative to another group *J* as the ratio of the CEMPs of the groups:

$$RMR_{I\ vs.J} = \frac{\sum COV_i^{mort} = 1}{\sum COV_i^{mort} = 0} / \frac{\sum COV_j^{mort} = 1}{\sum COV_j^{mort} = 0}$$

Note that the RMR is an odds ratio, also obtainable from logistic regression. We both compute RMRs directly and conduct multivariate logistic regression analysis to measure RMR vs. unvaccinated for groups defined by vaccination status (1, 2-, or 3-doses). Besides indicators for vaccine status (VS_i), the regression predictors $\boldsymbol{X}_i$ include days since most-recent dose (minus 14 days); age, age^2, zip-code-level socio-economic status (zip-SES), measured using the Graham Social Deprivation Index,[5] gender, race/ethnicity, education level, marital status, and military veteran status. The regression model is:

$$Prob(COV_i^{mort} = 1) = \boldsymbol{f}(\boldsymbol{VS}_i, \boldsymbol{X}_i)$$

To assess differences in underlying health between two groups, proxied by their mortality rate from other natural causes, we need to estimate population. We use population estimates for

2020 from the American Community Survey. We measure the number of people receiving 1, 2, or 3 vaccine doses and assume the remaining population is unvaccinated. We define non-Covid-NMR for a population which received *v* doses of vaccine type *i*, in time period *t*, as:

$$NonCovidNMR_{ivt} = \frac{\sum natural\ non-COVID-19\ deaths_{ivt}}{Population_{ivt}}$$

Time Periods and Booster Rollout

Boosters were first authorized by the Centers for Disease Control and Prevention (CDC) on September 24, 2021, initially for ages 60+ and healthcare workers and others in high -risk occupations. The CDC authorized boosters for all adults on November 19, 2021, but recommended them only for ages 50+.[5] Finally, on November 29, 2021, the CDC recommended boosters for all adults.[6]

Details on Younger, Fully Vaccinated COVID-19 Decedents

We found only one two-dose vaccinated person who died of COVID-19 in the age range from 18-49: A 35-year-old Black woman with sickle cell anemia, hemochromatosis, and pulmonary hypertension; vaccinated with Pfizer in April and May 2021; who died in January 2022.

Vaccination Counts and Rates

We obtained data on vaccinated individuals in Milwaukee County, including date of each vaccine dose, and vaccine type for each dose from the Wisconsin Immunization Registry. Table S2 provides summary data on vaccinated adults in Milwaukee, how many doses they received, and which vaccine they received. Overall, through March 31, 2022, around 75% of the adult population received at least one dose, and 70% were fully vaccinated (defined as one J&J dose or two mRNA doses). Of two-dose recipients, 56% received a third dose. These percentages are broadly in line with national averages.

Vaccine uptake was faster and more complete among those aged 60+, highest for ages 60-79, and slower and less complete at younger ages (Figure S3). Three-dose percentages (conditional on receiving two doses) rise with age, but are similar for ages 60-79 and 80+.

Selection Effects for One-Dose Recipients

In text Table 1, we report Non-COVID Natural Mortality Rates (Non-Covid-NMRs) for the unvaccinated, and for 2-dose and 3-dose vaccinees. The lower Non-Covid-NMRs for the vaccinated provide evidence of selection effects, in which vaccinated persons are, on average, healthier than the unvaccinated, reflected in lower Non-Covid-NMRs. In Table S3, we provide similar information for one-dose vaccinees. Younger one-dose vaccinees have lower Non-Covid-NMRs than the unvaccinated, but this is not true for the elderly.

Table S3 also reports combined results for ages 18-59 and ages 60+; in contrast, text Table 1 reports only results for finer age ranges. We report the combined results to show that reporting

[5] CDC Press Release (Nov. 19, 2021); Mandavilli (2021).

[6] CDC Press Release (Nov. 29, 2021).

this way leads to bias due to compositional effects, which arise because the proportion of the population which is vaccinated varies with age. For example, in April-June 2021, the ratio of Non-COVID NMR for 2-dose recipients to unvaccinated is 5,4% for ages 18-39 and 39.4% for ages 40-59, yet 44.4% for the combined ages range 18-59.

Confidence Intervals for Text Table 2

Table S4 provides 95% confidence intervals (CIs) for the relative mortality risk (RMR) values reported in text Table 2.

Additional Results for Text Table 2

Table S5 provides additional results, to supplement those reported in text Table 2. First, the table reports results separately for 1Q-2022 and 2Q-2022, which are combined in the text. As Table S4 indicates, there were too few COVID-19 deaths in 2Q-2022 to derive meaningful results for this period by itself. In the text, we therefore combined it with 1Q-2022. Second, the table reports RMR for two-doses versus one-dose and three-versus-two-doses for ages 18-59 and ages 60+. The text reports this information only for all ages combined.

Extended Multivariate Logit Results

In text Table 3 we report multivariate logit results for two-dose and three-dose vaccinees. In Table S6, we report additional results: for one-dose vaccinees, and for finer age groups.

Mortality-Weighted versus Population-Weighted Results

The overall CEMP and RMR estimates for broad age groups, presented in the bottom rows of text Table 2, are effectively weighted by the number of natural, non-COVID decedents in each cell. This weighting feels appropriate to us, because it weights RMR toward the people who are most affected by COVID-19. However, one could also weight RMR estimates by population, and this is typically done in other studies. We therefore use population weights in Table S7 for greater comparability to other studies. These estimates are generally, although not always, lower than our mortality-weighted estimates.

Defining the Immune-Compromised

In the results in text, we exclude persons known to be immune-compromised, defined as: (i) decedents whose death records indicate a solid organ transplant; (ii) decedents whose death records state that they were immune-compromised; and (iii) persons who received a third vaccine dose on or before September 24, 2022, the first date when a third dose was authorized for the non-immune-compromised. The standard CDC recommendation for full vaccination included a third "primary" dose; for these persons, the first booster dose would be a fourth dose. The booster studies by Arbel et. al (2021) and Bar-On et. al (2021) also exclude early third-dose recipients.

We report results without excluding the immune-compromised in Table S8. As expected, RMR values are generally lower. This effect is more pronounced for three-dose recipients, for whom three-dose RMR versus the unvaccinated is 16.4% in 4Q-2021 and 12.0% in 1Q-2022, versus 8.4% and 8.7% in text Table 2.

Conversely, our approach to excluding the immune-compromised is likely to undercount them, and thus overstate RMR. Therefore, as a robustness check, in Table S9, we also exclude all decedents whose death certificates refer to cancer, many of whom will be immune-compromised to varying degrees due to cancer treatment. As expected, RMR values decline, but only modestly. For example, two-dose RMR versus the unvaccinated is (12.4%, 17.5%, 22.6%, 34.7%) across the four sample quarters, versus (15.5%, 19.0%, 22.9%, 36.0%) in text Table 1; and three-dose RMR versus the unvaccinated for the two available quarters is (7.2%, 8.3%) versus (8.4%, 8.7%) in text Table 2.

J&J Vaccinees

We measure RMR and VE based on number of doses, thus treating one J&J dose as equivalent to one mRNA dose. This is consistent with our reading of the literature comparing J&J to the mRNA vaccines (e.g., Lin et. al, 2022). As a robustness check, in Table S10, we exclude J&J vaccinees (around 4% of all vaccinees, see Table S2). Results are similar to those in text Table 1, with RMRs sometimes modestly higher and sometimes modestly lower. For example, two-dose RMR versus the unvaccinated is (15.5%, 19.0%, 23.0%, 37.5%) across the four sample quarters, versus (15.5%, 19.0%, 22.9%, 36.0%) in text Table 2. Three dose RMR versus the unvaccinated is unaffected because none of the J&J recipients in our sample received three J&J doses; the small number who received three doses received an mRNA third dose.

In unreported results, we confirm that RMR averaged over our sample period is similar for one-dose J&J recipients and one-dose mRNA recipients, versus the unvaccinated (results available from the authors on request).

Lag Period Between Vaccine Administration and Effectiveness Against Death

We treat a vaccine dose as effective against mortality 30 days after receipt. In Table S10, we instead use a 14-day delay period. This shorter period is often used on other studies, and may be appropriate if the outcome of interest is infection. We prefer the longer, 30-day period , to account for both the lag between vaccine administration and full efficacy against infection, and the lag between infection and death. Other studies suggest a mean of 18 days from symptom onset (itself a few days after infection) to death for COVID-19 decedents.[7]

The shorter delay period has the effect of assigning deaths between 14 and 30 days after last dose to the unvaccinated or to a less-vaccinated group, and thus will lead to higher RMRs for between-group comparisons. Two-dose RMR versus the unvaccinated for all ages is somewhat higher than in text Table 1, especially in the first period, at (15.7%, 17.8%, 21.9%, 37.0%) across the four sample periods, versus (10.6%, 17.3%, 21.1%, and 36.2%) in text Table 2. Three-dose RMR versus the unvaccinated for the two available quarters is similar, at (8.0%, 10.8%) with the 14-day lag, versus (7.7%, 11.0%) in text Table 2.

Failure to allow a sufficient period between vaccine administration and death can lead to misleading results. An extreme example is a U.K. study which reported 98.7% VE for a Pfizer

[7] Yang et. al (2020), Marschner (2021).

booster against death [CI 97.4, 99.4].[8] However, this study measured death only over 14-34 days after booster receipt.

Results by Gender

In Table S12, we present results, similar to text Table 2, separately for men and women. Two-dose RMR versus the unvaccinated is (16.9%, 17.9%, 20.2%, 51.0%) across the four sample quarters for men (all ages), versus (6.0%, 17.0%, 22.2%, 23.8%) for women; note the very high RMR for men in 1H-2022, which is present for both ages 18-59 and ages 60+. Three-dose RMR versus the unvaccinated for the two available quarters is (5.7%, 14.0%) for men versus (9.6%, 8.6%) for women; RMR is again lower for women in the Omicron period. The Omicron period differences in RMRs for men versus women warrant further study.

Results for White versus Minority Decedents

In Table S13, we present results, similar to text Table 3, separately for non-Hispanic Whites, and minorities (all other persons). Sample size is too small to permit further decomposition of the results. Two-dose RMR versus the unvaccinated is (12.2%, 19.9%, 23.7%, 33.9%) across the four sample quarters for Whites (all ages), versus (12.6%, 18.8%, 19.7%, 40.2%) for minorities; thus, sometimes higher for Whites but sometimes lower. Three-dose RMR versus the unvaccinated for the two available quarters is (3.7%, 12.5%) for Whites versus (24.4%, 8.2%) for minorities. Although the small number of COVID-19 deaths among two- and three-dose recipients produce noisy estimates, there is no evidence of differences between White and minority RMRs.

Confidence Intervals for Text Table 3

Table S14 provides 95% confidence intervals (CIs) for the relative non-COVID-19 natural mortality rates (NCNMRs) reported in text Table 3.

RMRs Measured Using COVID-19 Mortality Rates Rather than CEMP

Table S15 provides results for RMRs measured using the COVID-19 Mortality Rate (CovidMR) as the outcome instead of CEMP, and thus measured without controlling for selection effects with regard to who gets vaccinated. These RMRs are comparable to other studies, which do not control for these selection effects. As expected, given the selection effects shown in text table 3, RMRs based on CovidMR are substantially lower than those reported in the text, and thus closer to those reported in other studies. For ages 60+, Two-dose RMR versus the unvaccinated is (8.0%, 10.9%, 17.5%, 23.9%) across the four sample quarters using CovidMR as the outcome, versus (16.9%, 24.4%, 30.0%, 32.4%) in text Table 1 (with CEMP as the outcome). Three-dose RMR versus the unvaccinated for ages 60+ for the two available quarters is (2.0%, 4.1%) using CovidMR as the outcome versus (8.4%, 8.7%) in text Table 1 (with CEMP as the outcome).

[8] Andrews et. al (2022).

The difference between RMRs with CEMP as the outcome, and RMRs with CovidMR as the outcome reflects selection effects. These are large (roughly 2:1) for two-dose versus unvaccinated, and roughly 3:1 for three-dose versus unvaccinated.

Compositional Effects for Full Sample Estimates

The bottom rows of Table S15 show results for all ages. These rows should be ignored, and are presented to show the importance of compositional effects when averaging across broad age groups, where: (i) older people are more likely to be vaccinated; and (ii) older people are more likely to die of COVID-19, controlling for vaccination status. One advantage of using CEMP as the outcome measure, rather than COVID-19 mortality rates, is that CEMP varies less with age and thus is less prone to compositional effects.

Evidence for Time-Varying Selection Effects and a Possibly Culling Effect

In text Table 1, the ratios of Non-Covid-NMRs for two-dose and three-dose vaccinees varies with age group and also with time period. For ages 60-79 and 80+, these ratios are higher in the Omicron period than earlier. We investigate that pattern, and possible explanations, in this section. Figure S8 reports monthly data for ages 80+ (Panel A) and ages 60-79 (Panel B), for total natural mortality (including COVID-19 deaths) for the unvaccinated (solid orange line), non-Covid-NMR for the unvaccinated (dashed orange line), and similar data (solid and dashed blue lines) for two- or three-dose vaccinees. Results for two- and three-dose vaccinees are combined; one-dose vaccinees and the immune-compromised are dropped from the sample.

For ages 80+, the gap between Non-Covid-NMR for unvaccinated versus vaccinated shrinks over time, and turns negative in 2022. For ages 60-79, this gap is reasonably stable for most of our sample period, but shrinks in 2Q-2022. There are at least three distinct reasons that, taken together, could explain these shrinking ratios:

(i) *Time-varying selection*. The nature of selection could change over time. It is plausible that the especially health-conscious and healthy get vaccinated first, and others follow more slowly. As more people become vaccinated, the Non-Covid NMR ratio for the unvaccinated will fall, relative to the vaccinated, because less healthy persons are moving from the unvaccinated pool to the vaccinated pool.

(ii) *Culling effect*. Some sick people, especially the frail elderly, were likely to die soon, of something. Covid pushed them over the edge, but they would otherwise have died of something else. Thus, especially soon after a peak in Covid deaths, one could see a fall in non-Covid natural deaths among the unvaccinated elderly.

(iii) *Adverse vaccine effect*. Getting vaccinated could be a shock to the system, especially for the frail elderly, that could lead to a medium-term rise in Non-Covid natural mortality and thus in Non-Covid NMR. This might not be captured by studies of vaccine side effects, most of which study short-term effects.

We can use the time pattern of the changes in relative Non-Covid-NMR to assess the plausibility and likely magnitude of each reason.

(i) *Time-varying selection*. This effect is consistent with the observed time patterns, but seems unlikely to be a full explanation, especially in 1H-2022. While booster percentages continued to rise in 1H-2022, total vaccination percentages for persons aged 60+ rose only modestly relative to late 2021(Figure S3). By this time, most people in these age groups had decided to get vaccinated, or not.

(ii) A culling effect would imply that Non-COVID-NMR for the unvaccinated relative to the vaccinated would fall after the peak in COVID mortality in late 2021 and early 2022. This is observed for both age groups.

(iii) *Adverse vaccine effect*. A vaccine effect on non-Covid-NMR for the vaccinated would be most likely soon after vaccination, and thus soon after the initial vaccination wave in early 2021, or perhaps after the initial wave of booster vaccination in 4Q-2021, likely followed by a dip after the initial surge. This is not observed in 2Q-2021, in the period soon after initial vaccination, For booster doses in late 2021, the dip in November 2021 for ages 80+ is inconsistent with a near-term effect of the booster dose on non-Covid-NMR.

Taking these three explanations together, we tentatively conclude; (i) time-varying selection is plausible, but is not likely to be the whole story; (ii) there is evidence for a culling effect shortly after periods of peak COVID-19 mortality; and (iii) there is no evidence for a strong adverse vaccine effect.

Note finally that Figure S8 provides confirmatory evidence for the value of vaccines. During the peak COVID period of late 2021-early 2022, the gap between total natural mortality (solid lines) and non-Covid-NMR (dashed lines) is substantially larger for the unvaccinated, especially for ages 60-79.

Additional References for supplementary materials (not cited in the text)

Griffin, Jennifer B, Meredith Haddix, Phoebe Danza, Rebecca Fisher, Tae Hee Koo, Elizabeth Traub, Prabhu Gounder, Claire Jarashow, and Sharon Balter (2021), SARS-CoV-2 Infections and Hospitalizations Among Persons Aged ≥16 Years, by Vaccination Status — Los Angeles County, California, May 1–July 25, 2021, *Morbidity and Mortality Weekly Report* 70(34), 1170-1176.

Mandavilli, Apoorva (2021), C.D.C. Endorses Covid Vaccine Booster Shots for Al Adults, *New York Times* (Nov. 19).

Marschner, Ian C. (2021). Estimating age-specific COVID-19 fatality risk and time to death by comparing population diagnosis and death patterns: Australian data, *BMC Medical Research Methodology* 21: 126.

Piernas, Carmen, Martina Patone, Nerys M Astbury, Min Gao, Aziz Sheikh, Kamlesh Khunti, Manu Shankar-Hari, Sharon Dixon, Carol Coupland, Paul Aveyard, Julia Hippisley-Cox*, and Susan A Jebb (2022), Associations of BMI with COVID-19 vaccine uptake, vaccine effectiveness, and risk of severe COVID-19 outcomes after vaccination in England: a population-based cohort study, *Lancet Diabetes and Endocrinology* at https://doi.org/10.1016/ S2213-8587(22)00158-9.

Rezel-Potts, Emma, Abdel Douiri, Xiaohui Sun, Phillip J. Chowienczyk, Ajay M. Shah, and Martin C. Gulliford (2022), Cardiometabolic outcomes up to 12 months after COVID-19 infection. A matched cohort study in the UK, *PLoS*, https://doi.org/10.1371/journal.pmed.1004052.

Yang, Xiaobo, Yuan Yu, Jioqian Xu, Huaqing Shu, Jia'an Xia*, Hong Liu, Yongran Wu, Lu Zhang, Zhui Yu, Minghao Fang, Ting Yu, Yaxin Wang, Shangwen Pan, Xiaojing Zou, Shiying Yuan, and You Shang (2020), Clinical course and outcomes of Critically Ill patients with SARS-CoV-2 pneumonia in Wuhan, China: a single-centered, retrospective, observational study. *Lancet Respiratory Medicine* 8: 475–481.

Table S1. Comparison of COVID-19 deaths per text analysis, to ICD-10 codes

Table shows counts, for April 1, 2020, through June 30, 2022, 2022, of COVID-19 deaths determined from our text analysis to COVID-19 deaths, determined using ICD-10 codes (the code for COVID-19 cause of death is U07.1). ICD-10 codes are generated by NCHS, based on the text fields in the death certificates. Deaths from natural causes are all deaths except those due to accident, homicide, or suicide. **Panel A.** Milwaukee County. **Panel B.** Wisconsin (statewide**.**

Panel A. Milwaukee County

**Milwaukee County**	**COVID-19 by Text Analysis**	**non-COVID-19 by Text Analysis**	**Total**
COVID-19 by ICD-10 Code	1,351	18	**1,369**
non-COVID-19 by ICD-10 Code	583	19,831	**20,414**
Missing ICD-10 Code	0	46	**46**
**Total**	**1,934**	**19,895**	**21,829**
From Natural Causes			18,690

Panel B. Wisconsin (statewide)

**Wisconsin (statewide)**	**COVID-19 by Text Analysis**	**non-COVID-19 by Text Analysis**	**Total**
COVID-19 by ICD-10 Code	11,303	209	**11,512**
non-COVID-19 by ICD-10 Code	1,292	122,494	**123,786**
Missing ICD-10 Code	0	188	**188**
**Total**	**12,595**	**122,891**	**135,486**
From Natural Causes			122,870

Table S2. Summary Statistics on Vaccination Status

Table provides summary information on vaccine doses for adults vaccinated in Milwaukee County, through June 30, 2022. Vaccine type for fully vaccinated persons is based on first two doses. Includes immune-compromised persons. For two-dose-recipients, breakdown by vaccine type is based on the first dose. The estimated Milwaukee adult population used to calculate percentage was 721,518 people.

Number of Doses	Vaccine Type	No. of People	% of pop.
Exactly 1	Janssen	19,000	2.6%
	Moderna	11,264	1.6%
	Pfizer	21,436	3.0%
	All 1 dose	51,700	7.2%
Exactly 2	Moderna Only	64,186	8.9%
	Pfizer Only	124,879	17.3%
	J&J Only	2,189	0.3%
	Mixed mRNA	1,018	0.1%
	Mixed J&J and mRNA	12,890	1.8%
	All 2 doses	205,162	28.4%
Exactly 3	Moderna Only	74,929	10.4%
	Pfizer Only	130,134	18.0%
	Mixed mRNA	23,357	3.2%
	Mixed J&J and mRNA	426	0.1%
	All 3 doses	228,846	31.7%
4+ (max 7)	All 4 doses	56,444	7.8%
**Total**		**542,152**	75.1%

Table S3. Non-Covid Natural Mortality Rate: 1-, 2-, and 3-Dose Recipients

Sample is same as text Table 1. Table shows Non-COVID Natural Mortality Rate (NCNMR) and relative NCNMR versus the unvaccinated, for people vaccinated with 1 dose, 2 doses, or 3 doses. Rates for unvaccinated, 2-dose vaccinees, and 3-dose vaccinees are same as text Table 1; this table also includes one-dose vaccinees, who were omitted from the text to simplify the text presentation. NCNMR is defined as the number of Non-COVID-19 natural deaths occurring among persons within the indicated age groups with the indicated vaccination status over the indicated period, divided by the estimated population of people in the same age group, vaccination status, and time period. The bottom two sets of rows sum results from the upper rows across broader age groups.

	**Time period**	**April-June 2021 (Alpha)**			**Jul-Sep 2021 (Delta no Booster)**			**Oct-Dec 2021 (Delta, With Booster)**				**Jan-June 2022 (Omicron)**			
Age	Measure	Unvax	1 dose	2 doses	Unvax	1 dose	2 doses	Unvax	1 dose	2 doses	3 doses	Unvax	1 dose	2 doses	3 doses
18-39	Non-Covid Natural MR	0.016%	0.011%	0.001%	0.022%	0.023%	0.007%	0.021%	0.010%	0.008%	0.000%	0.031%	0.022%	0.015%	0.008%
	NCNMR ratio to unvax		**70.4%**	**5.4%**		**104.7%**	**34.4%**		**49.5%**	**36.9%**	**0.0%**		**70.8%**	**49.0%**	**27.1%**
40-59	Non-Covid Natural MR	0.133%	0.103%	0.053%	0.138%	0.138%	0.046%	0.155%	0.193%	0.077%	0.056%	0.313%	0.315%	0.149%	0.110%
	NCNMR ratio to unvax		**77.0%**	**39.4%**		**100.4%**	**33.2%**		**124.6%**	**49.9%**	**36.4%**		**100.7%**	**47.5%**	**35.0%**
60-79	Non-Covid Natural MR	0.852%	0.507%	0.279%	0.930%	0.743%	0.298%	0.984%	0.705%	0.438%	0.198%	1.695%	0.859%	1.194%	0.599%
	NCNMR ratio to unvax		**59.5%**	**32.7%**		**79.9%**	**32.0%**		**71.7%**	**44.5%**	**20.1%**		**50.7%**	**70.4%**	**35.4%**
80+	Non-Covid Natural MR	2.582%	2.531%	1.598%	1.988%	3.708%	1.875%	2.550%	3.735%	2.467%	1.032%	3.848%	4.707%	4.743%	4.085%
	NCNMR ratio to unvax		**98.0%**	**61.9%**		**186.5%**	**94.3%**		**146.5%**	**96.7%**	**40.5%**		**122.3%**	**123.3%**	**106.2%**
Total 18-59	Non-Covid Natural MR	0.059%	0.054%	0.026%	0.063%	0.076%	0.026%	0.067%	0.089%	0.039%	0.010%	0.130%	0.134%	0.069%	0.062%
	NCNMR ratio to unvax		**92.0%**	**44.4%**		**121.1%**	**41.1%**		**132.8%**	**58.3%**	**14.7%**		**103.3%**	**53.4%**	**48.0%**
Total 60+	Non-Covid Natural MR	1.190%	0.742%	0.529%	1.173%	1.150%	0.566%	1.369%	1.136%	0.771%	0.339%	2.223%	1.496%	1.755%	1.180%
	NCNMR ratio to unvax		**62.4%**	**44.5%**		**98.1%**	**48.3%**		**82.9%**	**56.3%**	**24.8%**		**67.3%**	**79.0%**	**53.1%**

Table S4. Point Estimates and Confidence Intervals for RMR Estimates, Including One-Dose Vaccinees

Table reports 95% confidence intervals (Cis) for the RMR levels reported in text Table 2, plus RMRs and Cis for one-dose vaccinees, and for two-versus-one-dose vaccinees. The estimates and confidence intervals are produced by logistic regressions (one for each calendar quarter and reference vaccination group) with an outcome variable equal to 1 for decedents who died of COVID-19 and 0 for decedents who died of other natural causes. The regressions use as predictors a set of indicators for vaccination status (1 dose, 2 doses, or 3 doses), with unvaccinated as the omitted category in the RMR estimates vs. unvaccinated, 1-dose as the omitted category for the RMR estimates vs. 1 dose, and 2-doses as the omitted category for the RMR estimates vs. 2-doses.

		1 dose		2 doses		3 doses	
Period	Remaining Risk	Estimate	95% CI	Estimate	95% CI	Estimate	95% CI
**Ages 18-59**							
Apr-Jun 2021	Vs. unvaccinated	131.9%	[34.3%, 507.1%]	0.0%			
Jul-Sep 2021	Vs. unvaccinated	48.9%	[18.0%, 132.6%]	6.2%	[0.8%, 45.8%]		
Oct-Dec 2021	Vs. unvaccinated	26.7%	[11.5%, 62.1%]	3.3%	[0.8%, 13.8%]	0.0%	
Jan-Mar 2022	Vs. unvaccinated	24.6%	[5.7%, 106.2%]	42.7%	[18.1%, 100.8%]	0.0%	
**Ages 60+**							
Apr-Jun 2021	Vs. unvaccinated	39.8%	[13.9%, 113.9%]	11.1%	[3.4%, 36.6%]		
Jul-Sep 2021	Vs. unvaccinated	62.4%	[33.6%, 115.9%]	20.8%	[13.1%, 33.2%]		
Oct-Dec 2021	Vs. unvaccinated	28.9%	[15.6%, 53.6%]	27.6%	[20.2%, 37.7%]	9.5%	[3.0%, 30.3%]
Jan-Mar 2022	Vs. unvaccinated	49.9%	[28.5%, 87.3%]	34.2%	[24.6%, 47.8%]	10.8%	[7.2%, 16.4%]
**Ages 18+**							
Apr-Jun 2021	Vs. unvaccinated	56.8%	[25.2%, 128.1%]	10.6%	[3.3%, 34.3%]		
	Vs. 1 dose			18.6%	[4.8%, 72.5%]		
	Vs. 2 doses						
Jul-Sep 2021	Vs. unvaccinated	57.8%	[34.2%, 97.6%]	17.3%	[11.2%, 26.8%]		
	Vs. 1 dose			30.0%	[16.1%, 55.8%]		
	Vs. 2 doses						
Oct-Dec 2021	Vs. unvaccinated	28.9%	[17.6%, 47.4%]	21.1%	[15.8%, 28.2%]	7.7%	[2.4%, 24.6%]
	Vs. 1 dose			73.0%	[42.9%, 124.2%]	26.7%	[7.7%, 92.4%]
	Vs. 2 doses					36.6%	[11.3%, 118.3%]
Jan-Mar 2022	Vs. unvaccinated	43.2%	[25.7%, 72.7%]	36.2%	[26.5%, 49.3%]	11.0%	[7.3%, 16.6%]
	Vs. 1 dose			83.7%	[47.8%, 146.5%]	25.5%	[13.7%, 47.5%]
	Vs. 2 doses					30.5%	[19.3%, 48.2%]

Table S5. Vaccine Effectiveness (VE) by Age Group and Time Period: Additional Details

Table is similar to text Table 2 but provides additional details: (i) the table reports results separately for 1Q-2022 and 2Q-2022, which are combined in the text; (ii) reports results for one-dose vaccinees; and (iii) reports RMR for two-doses versus-one-dose and three-versus-two-doses for broader age groups.

Age		Apr-Jun 2021			July-Sep 2021			Oct-Dec 2021				Jan-Mar 2022				Apr-Jun 2022			
Group	Measure	0 doses	1 dose	2 doses	0 doses	1 dose	2 doses	0 doses	1 dose	2 doses	3 doses	0 doses	1 dose	2 doses	3 doses	0 doses	1 dose	2 doses	3 doses
18-39	Covid deaths	2	0	0	7	0	0	16	0	0	0	3	0	1	0	1	0	0	0
	Other natural deaths	31	4	1	32	7	9	26	3	11	0	19	3	11	2	13	3	7	2
	CEMP	6.5%	0.0%	0.0%	21.9%	0.0%	0.0%	61.5%	0.0%	0.0%	NA	15.8%	0.0%	9.1%	0.0%	7.7%	0.0%	0.0%	0.0%
	RMR vs. Unvaccinated		0.0%	0.0%		0.0%	0.0%		0.0%	0.0%	NA		0.0%	57.6%	0.0%		0.0%	0.0%	0.0%
40-59	Covid deaths	7	3	0	31	5	1	57	7	2	0	23	2	6	0	0	0	0	0
	Other natural deaths	147	41	27	113	32	53	102	43	95	3	97	38	50	29	80	19	59	55
	CEMP	4.8%	7.3%	0.0%	27.4%	15.6%	1.9%	55.9%	16.3%	2.1%	0.0%	23.7%	5.3%	12.0%	0.0%	0.0%	0.0%	0.0%	0.0%
	RMR vs. Unvaccinated		153.7%	0.0%		57.0%	6.9%		29.1%	3.8%	0.0%		22.2%	50.6%	0.0%		0.0%	0.0%	0.0%
60-79	Covid deaths	26	3	1	49	7	12	95	6	30	1	88	8	23	7	2	0	2	3
	Other natural deaths	367	114	226	297	76	338	266	74	380	45	245	52	252	239	171	22	175	298
	CEMP	7.1%	2.6%	0.4%	16.5%	9.2%	3.6%	35.7%	8.1%	7.9%	2.2%	35.9%	15.4%	9.1%	2.9%	1.2%	0.0%	1.1%	1.0%
	RMR vs. Unvaccinated		37.1%	6.2%		55.8%	21.5%		22.7%	22.1%	6.2%		42.8%	25.4%	8.2%		0.0%	3.2%	2.8%
80+	Covid deaths	6	1	2	26	6	13	49	6	37	2	51	7	26	12	5	0	1	6
	Other natural deaths	273	87	313	189	59	439	226	68	447	63	174	41	215	368	133	34	110	374
	CEMP	2.2%	1.1%	0.6%	13.8%	10.2%	3.0%	21.7%	8.8%	8.3%	3.2%	29.3%	17.1%	12.1%	3.3%	3.8%	0.0%	0.9%	1.6%
	RMR vs. Unvaccinated		52.3%	29.1%		73.9%	21.5%		40.7%	38.2%	14.6%		58.2%	41.3%	11.1%		0.0%	3.1%	5.5%
**Total 18-59**	Covid deaths	9	3	0	38	5	1	73	7	2	0	26	2	7	0	1	0	0	0
	CEMP	5.1%	6.7%	0.0%	26.2%	12.8%	1.6%	57.0%	15.2%	1.9%	0.0%	22.4%	4.9%	11.5%	0.0%	1.1%	0.0%	0.0%	0.0%
	RMR vs. Unvaccinated		131.9%	0.0%		48.9%	6.2%		26.7%	3.3%	0.0%		21.8%	51.2%	0.0%		0.0%	0.0%	0.0%
	RMR (versus 1 dose)			0.0%			12.6%			12.4%	0.0%			235.2%	0.0%			NA	NA
	RMR (versus 2 doses)										0.0%				0.0%				NA
**Total 60+**	Covid deaths	32	4	3	75	13	25	144	12	67	3	139	15	49	19	7	0	3	9
	CEMP	5.0%	2.0%	0.6%	15.4%	9.6%	3.2%	29.3%	8.5%	8.1%	2.8%	33.2%	16.1%	10.5%	3.1%	2.3%	0.0%	1.1%	1.3%
	RMR vs. Unvaccinated		39.8%	11.1%		62.4%	20.8%		28.9%	27.7%	9.5%		48.6%	31.6%	9.4%		0.0%	3.2%	4.0%
	RMR (versus 1 dose)			28.0%			33.4%			95.9%	32.9%			65.1%	19.4%			NA	NA
	RMR (versus 2 doses)										34.3%				29.8%				127.2%
**Total**	**Covid deaths**	41	7	3	113	18	26	217	19	69	3	165	17	56	19	8	0	3	9
	**CEMP**	5.0%	2.8%	0.5%	17.9%	10.3%	3.1%	35.0%	10.1%	7.4%	2.7%	30.8%	12.7%	10.6%	3.0%	2.0%	0.0%	0.9%	1.2%
	**RMR (versus unvax)**		56.8%	10.6%		57.8%	17.3%		28.9%	21.1%	7.7%		41.1%	34.4%	9.7%		0.0%	2.8%	4.0%
	**RMR (versus 1 dose)**			18.6%			30.0%			73.2%	26.7%			83.6%	23.5%			NA	NA
	**RMR (versus 2 doses)**										36.5%				28.1%				144.4%

Table S6. Multivariate Logit Results for One-Dose Vaccinees and Finer Age Groups

Table is similar to text Table 3, but: (i) includes result for one-dose vaccinees; and (ii) uses finer age groups.

			1 Dose		2 Doses		3 Doses	
Sample	Period	Remaining Risk	Estimate	95% CI	Estimate	95% CI	Estimate	95% CI
**18-39**	Apr-Jun 2021	Vs. unvax	0%[na]		0%[na]		No booster	
	Jul-Sep 2021	Vs. unvax	0%[na]		0%[na]		No booster	
	Oct-Dec 2021	Vs. unvax	0%[na]		0%[na]	0%[na]	0% [na]	
		Vs. 2 doses					0% [na]	
	Jan-Jun 2022	Vs. unvax	0%[na]		38.5%	[0.7%, 2072.1%]	0% [na]	
		Vs. 2 doses					0% [na]	
**40-59**	Apr-Jun 2021	Vs. unvax	231.9%	[38.0%, 1414.5%]	0%[na]		No booster	
	Jul-Sep 2021	Vs. unvax	44.7%	[15.3%, 130.6%]	**4.8%*****	**[0.4%, 51.0%]**	No booster	
	Oct-Dec 2021	Vs. unvax	**16.5%*****	**[6.1%, 45.1%]**	**2.3%*****	**[0.6%, 9.1%]**	0% [na]	
		Vs. 2 doses					0% [na]	
	Jan-Jun 2022	Vs. unvax	30.0%	[6.9%, 131.4%]	37.1%***	[12.9%, 106.6%]	0% [na]	
		Vs. 2 doses					0% [na]	
**60-79**	Apr-Jun 2021	Vs. unvax	30.9%	[8.8%, 107.9%]	**5.5%****	**[0.8%, 39.8%]**	No booster	
	Jul-Sep 2021	Vs. unvax	55.2%	[22.7%, 134.0%]	**21.9%*****	**[10.7%, 44.9%]**	No booster	
	Oct-Dec 2021	Vs. unvax	**18.8%***	**[7.7%, 46.4%]**	**20.8%*****	**[13.2%, 32.8%]**	**6.2%****	**[0.8%, 47.2%]**
		Vs. 2 doses					33.7%	[3.8%, 296.4%]
	Jan-Jun 2022	Vs. unvax	**43.2%***	**[19.8%, 94.0%]**	**23.9%*****	**[15.0%, 38.2%]**	**8.1%*****	**[4.0%, 16.3%]**
		Vs. 2 doses					**33.1%****	**[15.0%, 72.9%]**
**80+**	Apr-Jun 2021	Vs. unvax	37.4%	[3.4%, 412.4%]	27.9%	[4.9%, 159.8%]	No booster	
	Jul-Sep 2021	Vs. unvax	67.6%	[25.7%, 177.6%]	21.3%***	[10.4%, 43.4%]	No booster	
	Oct-Dec 2021	Vs. unvax	43.1%	[17.1%, 108.7%]	**39.5%*****	**[24.0%, 65.1%]**	**14.9%****	**[3.7%, 60.1%]**
		Vs. 2 doses					36.9%	[9.1%, 149.6%]
	Jan-Jun 2022	Vs. unvax	53.1%	[22.5%, 125.6%]	**42.8%*****	**[25.9%, 70.7%]**	**14.4%*****	**[8.0%, 25.6%]**
		Vs. 2 doses					**30.1%****	**[16.0%, 56.5%]**

Table S7. Population-Weighted CEMP and RMR by Time Period

Table is similar to bottom part of text Table 2, but reports population-weighted CEMP and RMR instead of the mortality-weighted values reported in Table 1, for easier comparison to other papers that report population-weighted estimates. Sample is same as Text Table 2.

	April-Jun 2021 (Alpha)			Jul-Sep 2021 (Delta no Booster)			Oct-Dec 2021 (Delta, With Booster)				Jan-Jun 2022 (Omicron)			
Measure (Population weighted)	0 doses	1 dose	2 doses	0 doses	1 dose	2 doses	0 doses	1 dose	2 doses	3 doses	0 doses	1 dose	2 doses	3 doses
CEMP	5.84%	2.91%	0.12%	22.19%	7.29%	1.45%	52.71%	7.22%	2.64%	0.59%	14.75%	3.72%	5.73%	0.49%
RMR (versus unvax)		49.82%	2.02%		32.87%	6.53%		13.69%	5.00%	1.12%		25.19%	38.81%	3.29%
RMR (versus 1 dose)			4.06%			19.88%			36.53%	8.21%			154.07%	13.07%
RMR (versus 2 doses)										22.48%				8.48%

Table S8. CEMP and RMR by Time Period, Including Immune-Compromised

Table is similar to text Table 2, but sample includes immune-compromised persons.

Age		April-Jun 2021 (Alpha)			Jul-Sep 2021 (Delta no Booster)			Oct-Dec 2021 (Delta, With Booster)				Jan-Jun 2022 (Omicron)			
Group	Measure	0 doses	1 dose	2 doses	0 doses	1 dose	2 doses	0 doses	1 dose	2 doses	3 doses	0 doses	1 dose	2 doses	3 doses
18-39	Covid deaths	2	0	0	7	0	0	18	0	0	0	4	0	1	0
	Other natural deaths	31	4	1	33	7	9	26	3	11	1	32	6	19	5
	CEMP	6.5%	0.0%	0.0%	21.2%	0.0%	0.0%	69.2%	0.0%	0.0%	0.0%	12.5%	0.0%	5.3%	0.0%
	RMR vs. Unvaccinated		0%	0%		0%	0%		0%	0%	0%		0%	42%	0%
40-59	Covid deaths	8	3	0	32	5	2	58	7	3	0	23	2	8	2
	Other natural deaths	149	43	27	114	34	56	103	43	95	9	180	58	110	91
	CEMP	5.4%	7.0%	0.0%	28.1%	14.7%	3.6%	56.3%	16.3%	3.2%	0.0%	12.8%	3.4%	7.3%	2.2%
	RMR vs. Unvaccinated		129.9%	0.0%		52.4%	12.7%		28.9%	5.6%	0.0%		27.0%	56.9%	17.2%
60-79	Covid deaths	27	4	1	49	7	16	97	6	36	5	92	9	29	19
	Other natural deaths	372	117	227	303	77	351	270	74	387	66	418	75	434	607
	CEMP	7.3%	3.4%	0.4%	16.2%	9.1%	4.6%	35.9%	8.1%	9.3%	7.6%	22.0%	12.0%	6.7%	3.1%
	RMR vs. Unvaccinated		47.1%	6.1%		56.2%	28.2%		22.6%	25.9%	21.1%		54.5%	30.4%	14.2%
80+	Covid deaths	6	1	2	27	6	14	50	6	38	3	57	7	27	19
	Other natural deaths	274	87	315	192	59	442	227	68	453	71	309	75	326	775
	CEMP	2.2%	1.1%	0.6%	14.1%	10.2%	3.2%	22.0%	8.8%	8.4%	4.2%	18.4%	9.3%	8.3%	2.5%
	RMR vs. Unvaccinated		52.5%	29.0%		72.3%	22.5%		40.1%	38.1%	19.2%		50.6%	44.9%	13.3%
**Total**	Covid deaths	10	3	0	39	5	2	76	7	3	0	27	2	9	2
**18-59**	CEMP	5.6%	6.4%	0.0%	26.5%	12.2%	3.1%	58.9%	15.2%	2.8%	0.0%	12.7%	3.1%	7.0%	2.1%
	RMR vs. Unvaccinated		114.9%	0.0%		46.0%	11.6%		25.8%	4.8%	0.0%		24.5%	54.8%	16.4%
**Total**	Covid deaths	33	5	3	76	13	30	147	12	74	8	149	16	56	38
**60+**	CEMP	5.1%	2.5%	0.6%	15.4%	9.6%	3.8%	29.6%	8.5%	8.8%	5.8%	20.5%	10.7%	7.4%	2.7%
	RMR vs. Unvaccinated		48.0%	10.8%		62.3%	24.6%		28.6%	29.8%	19.7%		52.0%	36.0%	13.4%
**Total**	**Covid deaths**	**43**	**8**	**3**	**115**	**18**	**32**	**223**	**19**	**77**	**8**	**176**	**18**	**65**	**40**
	**CEMP**	**5.2%**	**3.2%**	**0.5%**	**17.9%**	**10.2%**	**3.7%**	**35.6%**	**10.1%**	**8.1%**	**5.4%**	**18.7%**	**8.4%**	**7.3%**	**2.7%**
	**RMR (versus unvax)**		**61.2%**	**10.1%**		**56.8%**	**20.8%**		**28.4%**	**22.8%**	**15.3%**		**44.9%**	**39.0%**	**14.4%**
	**RMR (versus 1 dose)**			**16.5%**			**36.7%**			**80.5%**	**53.8%**			**86.9%**	**32.2%**
	**RMR (versus 2 doses)**										**66.9%**				**37.0%**

Table S9. CEMP and RMR by Time Period, Excluding Immune-compromised and Decedents with Cancer

Table is similar to text Table 2, but sample excludes both known immune-compromised persons and persons with cancer indicated in death certificates, many of whom will be immune-compromised.

Age		**April-Jun 2021 (Alpha)**			**Jul-Sep 2021 (Delta no Booster)**			**Oct-Dec 2021 (Delta, With Booster)**				**Jan-Jun 2022 (Omicron)**			
Group	Measure	**0 doses**	**1 dose**	**2 doses**	**0 doses**	**1 dose**	**2 doses**	**0 doses**	**1 dose**	**2 doses**	**3 doses**	**0 doses**	**1 dose**	**2 doses**	**3 doses**
18-39	Covid deaths	2	0	0	7	0	0	16	0	0	0	4	0	1	0
	Other natural deaths	28	4	0	28	7	8	21	3	8	0	28	6	14	4
	CEMP	7.1%	0.0%	NA	25.0%	0.0%	0.0%	76.2%	0.0%	0.0%	NA	14.3%	0.0%	7.1%	0.0%
	RMR vs. Unvaccinated		0.0%	NA		0.0%	0.0%		0.0%	0.0%	NA		0.0%	50.0%	0.0%
40-59	Covid deaths	7	3	0	28	5	1	57	7	1	0	23	2	6	0
	Other natural deaths	107	33	23	80	25	44	80	36	73	2	133	45	81	62
	CEMP	6.5%	9.1%	0.0%	35.0%	20.0%	2.3%	71.3%	19.4%	1.4%	0.0%	17.3%	4.4%	7.4%	0.0%
	RMR vs. Unvaccinated		139.0%	0.0%		57.1%	6.5%		27.3%	1.9%	0.0%		25.7%	42.8%	0.0%
60-79	Covid deaths	24	3	1	45	5	10	84	6	27	1	82	8	19	8
	Other natural deaths	240	85	152	217	58	238	191	51	281	33	298	53	305	384
	CEMP	10.0%	3.5%	0.7%	20.7%	8.6%	4.2%	44.0%	11.8%	9.6%	3.0%	27.5%	15.1%	6.2%	2.1%
	RMR vs. Unvaccinated		35.3%	6.6%		41.6%	20.3%		26.8%	21.8%	6.9%		54.9%	22.6%	7.6%
80+	Covid deaths	6	1	1	23	6	13	42	6	33	1	52	5	23	16
	Other natural deaths	217	72	261	152	49	354	198	53	367	51	260	64	286	635
	CEMP	2.8%	1.4%	0.4%	15.1%	12.2%	3.7%	21.2%	11.3%	9.0%	2.0%	20.0%	7.8%	8.0%	2.5%
	RMR vs. Unvaccinated		50.2%	13.9%		80.9%	24.3%		53.4%	42.4%	9.2%		39.1%	40.2%	12.6%
**Total**	Covid deaths	9	3	0	35	5	1	73	7	1	0	27	2	7	0
**18-59**	CEMP	6.7%	8.1%	0.0%	32.4%	15.6%	1.9%	72.3%	17.9%	1.2%	0.0%	16.8%	3.9%	7.4%	0.0%
	RMR vs. Unvaccinated		121.6%	0.0%		48.2%	5.9%		24.8%	1.7%	0.0%		23.4%	43.9%	0.0%
**Total**	Covid deaths	30	4	2	68	11	23	126	12	60	2	134	13	42	24
**60+**	CEMP	6.6%	2.5%	0.5%	18.4%	10.3%	3.9%	32.4%	11.5%	9.3%	2.4%	24.0%	11.1%	7.1%	2.4%
	RMR vs. Unvaccinated		38.8%	7.4%		55.8%	21.1%		35.6%	28.6%	7.4%		46.3%	29.6%	9.8%
**Total**	**Covid deaths**	39	7	2	103	16	24	199	19	61	2	161	15	49	24
	**CEMP**	6.6%	3.6%	0.5%	21.6%	11.5%	3.7%	40.6%	13.3%	8.4%	2.3%	22.4%	8.9%	7.1%	2.2%
	**RMR (versus unvax)**		54.8%	7.0%		53.3%	17.3%		32.7%	20.6%	5.7%		39.9%	31.9%	9.9%
	**RMR (versus 1 dose)**			12.7%			32.4%			63.0%	17.5%			80.0%	24.8%
	**RMR (versus 2 doses)**										27.8%				31.0%

Table S10. CEMP and RMR by Time Period, Excluding J&J Vaccinees

Table is similar to text Table 2, but sample excludes J&J vaccine recipients.

Age		April-Jun 2021 (Alpha)			Jul-Sep 2021 (Delta no Booster)			Oct-Dec 2021 (Delta, With Booster)				Jan-Jun 2022 (Omicron)			
Group	Measure	0 doses	1 dose	2 doses	0 doses	1 dose	2 doses	0 doses	1 dose	2 doses	3 doses	0 doses	1 dose	2 doses	3 doses
18-39	Covid deaths	2	0	0	7	0	0	16	0	0	0	4	0	1	0
	Other natural deaths	31	3	1	32	4	9	26	1	11	0	32	3	17	4
	CEMP	6.5%	0.0%	0.0%	21.9%	0.0%	0.0%	61.5%	0.0%	0.0%	NA	12.5%	0.0%	5.9%	0.0%
	RMR vs. Unvaccinated		0%	0%		0%	0%		0%	0%	NA		0%	47%	0%
40-59	Covid deaths	7	2	0	31	2	1	57	4	2	0	23	0	6	0
	Other natural deaths	147	33	27	113	18	53	102	18	95	3	177	39	102	84
	CEMP	4.8%	6.1%	0.0%	27.4%	11.1%	1.9%	55.9%	22.2%	2.1%	0.0%	13.0%	0.0%	5.9%	0.0%
	RMR vs. Unvaccinated		127.3%	0.0%		40.5%	6.9%		39.8%	3.8%	0.0%		0.0%	45.3%	0.0%
60-79	Covid deaths	26	3	1	49	4	12	95	5	30	1	90	6	25	10
	Other natural deaths	367	105	226	297	49	338	266	36	378	45	416	47	407	537
	CEMP	7.1%	2.9%	0.4%	16.5%	8.2%	3.6%	35.7%	13.9%	7.9%	2.2%	21.6%	12.8%	6.1%	1.9%
	RMR vs. Unvaccinated		40.3%	6.2%		49.5%	21.5%		38.9%	22.2%	6.2%		59.0%	28.4%	8.6%
80+	Covid deaths	6	1	2	26	4	13	49	5	37	2	56	4	27	18
	Other natural deaths	273	81	313	189	38	439	226	34	447	63	307	54	308	742
	CEMP	2.2%	1.2%	0.6%	13.8%	10.5%	3.0%	21.7%	14.7%	8.3%	3.2%	18.2%	7.4%	8.8%	2.4%
	RMR vs. Unvaccinated		56.2%	29.1%		76.5%	21.5%		67.8%	38.2%	14.6%		40.6%	48.1%	13.3%
**Total 18-59**	Covid deaths	9	2	0	38	2	1	73	4	2	0	27	0	7	0
	CEMP	5.1%	5.6%	0.0%	26.2%	9.1%	1.6%	57.0%	21.1%	1.9%	0.0%	12.9%	0.0%	5.9%	0.0%
	RMR vs. Unvaccinated		109.9%	0.0%		34.7%	6.2%		36.9%	3.3%	0.0%		0.0%	45.5%	0.0%
**Total 60+**	Covid deaths	32	4	3	75	8	25	144	10	67	3	146	10	52	28
	CEMP	5.0%	2.2%	0.6%	15.4%	9.2%	3.2%	29.3%	14.3%	8.1%	2.8%	20.2%	9.9%	7.3%	2.2%
	RMR vs. Unvaccinated		43.0%	11.1%		59.6%	20.8%		48.8%	27.7%	9.5%		49.0%	36.0%	10.8%
**Total**	**Covid deaths**	41	6	3	113	10	26	217	14	69	3	173	10	59	28
	**CEMP**	5.0%	2.7%	0.5%	17.9%	9.2%	3.1%	35.0%	15.7%	7.4%	2.7%	18.6%	7.0%	7.1%	2.0%
	**RMR (versus unvax)**		53.9%	10.6%		51.2%	17.3%		44.9%	21.2%	7.7%		37.7%	38.1%	11.0%
	**RMR (versus 1 dose)**			19.6%			33.8%			47.1%	17.2%			101.2%	29.3%
	**RMR (versus 2 doses)**										36.5%				29.0%

Table S11. RMR by Time Period Using 14-day Minimum from Vaccination to Death

Table and sample are same as text Table 2, but we require a minimum of 14-days from last vaccination dose to death to count the last dose when classifying people by number of vaccine doses, instead of the 30-day minimum in the text.

Age		April-Jun 2021 (Alpha)			Jul-Sep 2021 (Delta no Booster)			Oct-Dec 2021 (Delta, With Booster)				Jan-Jun 2022 (Omicron)			
Group	Measure	0 doses	1 dose	2 doses	0 doses	1 dose	2 doses	0 doses	1 dose	2 doses	3 doses	0 doses	1 dose	2 doses	3 doses
18-39	Covid deaths	2	0	0	7	0	0	16	0	0	0	4	0	1	0
	Other natural deaths	31	4	1	32	6	10	26	3	11	0	32	6	16	6
	CEMP	6.5%	0.0%	0.0%	21.9%	0.0%	0.0%	61.5%	0.0%	0.0%	NA	12.5%	0.0%	6.3%	0.0%
	RMR vs. Unvaccinated		0%	0%		0%	0%		0%	0%	NA		0%	50%	0%
40-59	Covid deaths	7	3	0	31	5	1	57	7	2	0	23	2	6	0
	Other natural deaths	147	31	37	113	29	56	102	42	92	7	177	54	108	88
	CEMP	4.8%	9.7%	0.0%	27.4%	17.2%	1.8%	55.9%	16.7%	2.2%	0.0%	13.0%	3.7%	5.6%	0.0%
	RMR vs. Unvaccinated		203.2%	0.0%		62.8%	6.5%		29.8%	3.9%	0.0%		28.5%	42.8%	0.0%
60-79	Covid deaths	26	1	3	49	7	12	95	6	30	1	90	8	25	10
	Other natural deaths	367	84	256	297	73	341	266	70	357	72	416	69	416	553
	CEMP	7.1%	1.2%	1.2%	16.5%	9.6%	3.5%	35.7%	8.6%	8.4%	1.4%	21.6%	11.6%	6.0%	1.8%
	RMR vs. Unvaccinated		16.8%	16.5%		58.1%	21.3%		24.0%	23.5%	3.9%		53.6%	27.8%	8.4%
80+	Covid deaths	6	1	2	26	5	14	49	6	35	4	56	7	27	18
	Other natural deaths	273	60	340	189	58	440	226	62	416	100	307	73	318	751
	CEMP	2.2%	1.7%	0.6%	13.8%	8.6%	3.2%	21.7%	9.7%	8.4%	4.0%	18.2%	9.6%	8.5%	2.4%
	RMR vs. Unvaccinated		75.8%	26.8%		62.7%	23.1%		44.6%	38.8%	18.4%		52.6%	46.5%	13.1%
**Total**	Covid deaths	9	3	0	38	5	1	73	7	2	0	27	2	7	0
**18-59**	CEMP	5.1%	8.6%	0.0%	26.2%	14.3%	1.5%	57.0%	15.6%	1.9%	0.0%	12.9%	3.3%	5.6%	0.0%
	RMR vs. Unvaccinated		169.5%	0.0%		54.5%	5.8%		27.3%	3.4%	0.0%		25.8%	43.7%	0.0%
**Total**	Covid deaths	32	2	5	75	12	26	144	12	65	5	146	15	52	28
**60+**	CEMP	5.0%	1.4%	0.8%	15.4%	9.2%	3.3%	29.3%	9.1%	8.4%	2.9%	20.2%	10.6%	7.1%	2.1%
	RMR vs. Unvaccinated		27.8%	16.8%		59.4%	21.6%		31.1%	28.7%	9.9%		52.3%	35.1%	10.6%
**Total**	**Covid deaths**	41	5	5	113	17	27	217	19	67	5	173	17	59	28
	**CEMP**	5.0%	2.8%	0.8%	17.9%	10.2%	3.2%	35.0%	10.7%	7.6%	2.8%	18.6%	8.4%	6.9%	2.0%
	**RMR (versus unvax)**		55.7%	15.7%		57.2%	17.8%		30.7%	21.9%	8.0%		45.3%	37.0%	10.8%
	**RMR (versus 1 dose)**			28.2%			31.1%			71.3%	26.0%			81.7%	23.8%
	**RMR (versus 2 doses)**										36.5%				29.1%

Table S12. CEMP and RMR by Time Period and Gender

Table and sample are same as text Table 2, but we show results separately for men (**Panel A**) and women (**Panel B**)

Panel A. Male

Age		**April-Jun 2021 (Alpha)**			**Jul-Sep 2021 (Delta no Booster)**			**Oct-Dec 2021 (Delta, With Booster)**				**Jan-Jun 2022 (Omicron)**			
Group	Measure	**0 doses**	**1 dose**	**2 doses**	**0 doses**	**1 dose**	**2 doses**	**0 doses**	**1 dose**	**2 doses**	**3 doses**	**0 doses**	**1 dose**	**2 doses**	**3 doses**
18-39	Covid deaths	2	0	0	4	0	0	8	0	0	0	2	0	0	0
	Other natural deaths	17	1	1	18	4	5	16	2	6	0	19	4	13	4
	CEMP	11.8%	0.0%	0.0%	22.2%	0.0%	0.0%	50.0%	0.0%	0.0%	NA	10.5%	0.0%	0.0%	0.0%
	RMR vs. Unvaccinated		0%	0%		0%	0%		0%	0%	NA		0%	0%	0%
40-59	Covid deaths	3	3	0	20	2	0	30	7	1	0	10	2	4	0
	Other natural deaths	86	25	11	61	23	30	55	24	50	1	122	33	59	52
	CEMP	3.5%	12.0%	0.0%	32.8%	8.7%	0.0%	54.5%	29.2%	2.0%	0.0%	8.2%	6.1%	6.8%	0.0%
	RMR vs. Unvaccinated		344.0%	0.0%		26.5%	0.0%		53.5%	3.7%	0.0%		73.9%	82.7%	0.0%
60-79	Covid deaths	14	1	0	21	2	6	44	3	15	0	45	5	17	4
	Other natural deaths	205	62	107	177	37	179	141	38	198	22	246	42	222	293
	CEMP	6.8%	1.6%	0.0%	11.9%	5.4%	3.4%	31.2%	7.9%	7.6%	0.0%	18.3%	11.9%	7.7%	1.4%
	RMR vs. Unvaccinated		23.6%	0.0%		45.6%	28.3%		25.3%	24.3%	0.0%		65.1%	41.9%	7.5%
80+	Covid deaths	2	0	2	15	1	6	32	1	17	1	32	5	16	11
	Other natural deaths	114	34	119	69	27	149	85	25	171	23	127	28	125	270
	CEMP	1.8%	0.0%	1.7%	21.7%	3.7%	4.0%	37.6%	4.0%	9.9%	4.3%	25.2%	17.9%	12.8%	4.1%
	RMR vs. Unvaccinated		0.0%	95.8%		17.0%	18.5%		10.6%	26.4%	11.5%		70.9%	50.8%	16.2%
**Total 18-59**	Covid deaths	5	3	0	24	2	0	38	7	1	0	12	2	4	0
	CEMP	4.9%	11.5%	0.0%	30.4%	7.4%	0.0%	53.5%	26.9%	1.8%	0.0%	8.5%	5.4%	5.6%	0.0%
	RMR vs. Unvaccinated		237.7%	0.0%		24.4%	0.0%		50.3%	3.3%	0.0%		63.5%	65.3%	0.0%
**Total 60+**	Covid deaths	16	1	2	36	3	12	76	4	32	1	77	10	33	15
	CEMP	5.0%	1.0%	0.9%	14.6%	4.7%	3.7%	33.6%	6.3%	8.7%	2.2%	20.6%	14.3%	9.5%	2.7%
	RMR vs. Unvaccinated		20.8%	17.6%		32.0%	25.0%		18.9%	25.8%	6.6%		69.2%	46.1%	12.9%
**Total**	**Covid deaths**	21	4	2	60	5	12	114	11	33	1	89	12	37	15
	**CEMP**	5.0%	3.3%	0.8%	18.5%	5.5%	3.3%	38.4%	12.4%	7.8%	2.2%	17.3%	11.2%	8.8%	2.4%
	**RMR (versus unvax)**		65.9%	16.9%		29.8%	17.9%		32.2%	20.2%	5.7%		64.8%	51.0%	14.0%
	**RMR (versus 1 dose)**			25.6%			60.2%			62.8%	17.6%			78.7%	21.6%
	**RMR (versus 2 doses)**										28.0%				27.4%

Panel B. Female

Age Group	Measure	April-Jun 2021 (Alpha)			Jul-Sep 2021 (Delta no Booster)			Oct-Dec 2021 (Delta, With Booster)				Jan-Jun 2022 (Omicron)			
		**0 doses**	**1 dose**	**2 doses**	**0 doses**	**1 dose**	**2 doses**	**0 doses**	**1 dose**	**2 doses**	**3 doses**	**0 doses**	**1 dose**	**2 doses**	**3 doses**
18-39	Covid deaths	0	0	0	3	0	0	8	0	0	0	2	0	1	0
	Other natural deaths	14	3	0	14	3	4	10	1	5	0	13	2	5	0
	CEMP	0.0%	0.0%	NA	21.4%	0.0%	0.0%	80.0%	0.0%	0.0%	NA	15.4%	0.0%	20.0%	NA
	RMR vs. Unvaccinated		NA	NA		0%	0%		0%	0%	NA		0%	130%	NA
40-59	Covid deaths	4	0	0	11	3	1	27	0	1	0	13	0	2	0
	Other natural deaths	61	16	16	52	9	23	47	19	45	2	55	24	50	32
	CEMP	6.6%	0.0%	0.0%	21.2%	33.3%	4.3%	57.4%	0.0%	2.2%	0.0%	23.6%	0.0%	4.0%	0.0%
	RMR vs. Unvaccinated		0.0%	0.0%		157.6%	20.6%		0.0%	3.9%	0.0%		0.0%	16.9%	0.0%
60-79	Covid deaths	12	2	1	28	5	6	51	3	15	1	45	3	8	6
	Other natural deaths	162	52	119	120	39	159	125	36	182	23	170	32	205	244
	CEMP	7.4%	3.8%	0.8%	23.3%	12.8%	3.8%	40.8%	8.3%	8.2%	4.3%	26.5%	9.4%	3.9%	2.5%
	RMR vs. Unvaccinated		51.9%	11.3%		54.9%	16.2%		20.4%	20.2%	10.7%		35.4%	14.7%	9.3%
80+	Covid deaths	4	1	0	11	5	7	17	5	20	1	24	2	11	7
	Other natural deaths	159	53	194	120	32	290	141	43	276	40	180	47	200	472
	CEMP	2.5%	1.9%	0.0%	9.2%	15.6%	2.4%	12.1%	11.6%	7.2%	2.5%	13.3%	4.3%	5.5%	1.5%
	RMR vs. Unvaccinated		75.0%	0.0%		170.5%	26.3%		96.4%	60.1%	20.7%		31.9%	41.3%	11.1%
**Total 18-59**	Covid deaths	4	0	0	14	3	1	35	0	1	0	15	0	3	0
	CEMP	5.3%	0.0%	0.0%	21.2%	25.0%	3.7%	61.4%	0.0%	2.0%	0.0%	22.1%	0.0%	5.5%	0.0%
	RMR vs. Unvaccinated		0.0%	0.0%		117.9%	17.5%		0.0%	3.3%	0.0%		0.0%	24.7%	0.0%
**Total 60+**	Covid deaths	16	3	1	39	10	13	68	8	35	2	69	5	19	13
	CEMP	5.0%	2.9%	0.3%	16.3%	14.1%	2.9%	25.6%	10.1%	7.6%	3.2%	19.7%	6.3%	4.7%	1.8%
	RMR vs. Unvaccinated		57.3%	6.4%		86.7%	17.8%		39.6%	29.9%	12.4%		32.1%	23.8%	9.2%
**Total**	**Covid deaths**	20	3	1	53	13	14	103	8	36	2	84	5	22	13
	**CEMP**	5.1%	2.4%	0.3%	17.3%	15.7%	2.9%	31.9%	8.1%	7.1%	3.1%	20.1%	4.8%	4.8%	1.7%
	**RMR (versus unvax)**		47.9%	6.0%		90.4%	17.0%		25.3%	22.2%	9.6%		23.7%	23.8%	8.6%
	**RMR (versus 1 dose)**			12.6%			18.8%			87.7%	38.1%			100.4%	36.5%
	**RMR (versus 2 doses)**										43.4%				36.3%

Table S13. CEMP and RMR by Time Period and White vs. Minority

Table and sample are similar to text Table 1, but we show results separately for non-Hispanic Whites (**Panel A**) and all other race/ethnicities (**Panel B**)

Panel A. Non-Hispanic White

Age		**April-Jun 2021 (Alpha)**			**Jul-Sep 2021 (Delta no Booster)**			**Oct-Dec 2021 (Delta, With Booster)**				**Jan-Jun 2022 (Omicron)**			
Group	Measure	**0 doses**	**1 dose**	**2 doses**	**0 doses**	**1 dose**	**2 doses**	**0 doses**	**1 dose**	**2 doses**	**3 doses**	**0 doses**	**1 dose**	**2 doses**	**3 doses**
18-39	Covid deaths	1	0	0	1	0	0	2	0	0	0	2	0	0	0
	Other natural deaths	11	0	1	11	3	3	2	0	6	0	10	2	5	1
	CEMP	9.1%	NA	0.0%	9.1%	0.0%	0.0%	100.0%	NA	0.0%	NA	20.0%	0.0%	0.0%	0.0%
	RMR vs. Unvaccinated		NA	0.0%		0%	0%		NA	0%	NA		0%	0%	0%
40-59	Covid deaths	1	0	0	10	0	1	21	3	1	0	8	1	3	0
	Other natural deaths	56	16	15	36	18	29	44	17	44	2	67	21	53	47
	CEMP	1.8%	0.0%	0.0%	27.8%	0.0%	3.4%	47.7%	17.6%	2.3%	0.0%	11.9%	4.8%	5.7%	0.0%
	RMR vs. Unvaccinated		0.0%	0.0%		0.0%	12.4%		37.0%	4.8%	0.0%		39.9%	47.4%	0.0%
60-79	Covid deaths	11	0	0	21	5	5	44	3	19	0	50	2	11	6
	Other natural deaths	201	71	166	178	40	230	144	35	249	33	242	36	254	379
	CEMP	5.5%	0.0%	0.0%	11.8%	12.5%	2.2%	30.6%	8.6%	7.6%	0.0%	20.7%	5.6%	4.3%	1.6%
	RMR vs. Unvaccinated		0.0%	0.0%		106.0%	18.4%		28.1%	25.0%	0.0%		26.9%	21.0%	7.7%
80+	Covid deaths	4	1	2	16	3	11	35	5	28	1	31	7	18	17
	Other natural deaths	199	67	269	134	34	377	149	45	373	56	211	57	238	641
	CEMP	2.0%	1.5%	0.7%	11.9%	8.8%	2.9%	23.5%	11.1%	7.5%	1.8%	14.7%	12.3%	7.6%	2.7%
	RMR vs. Unvaccinated		74.3%	37.0%		73.9%	24.4%		47.3%	32.0%	7.6%		83.6%	51.5%	18.1%
**Total 18-59**	Covid deaths	2	0	0	11	0	1	23	3	1	0	10	1	3	0
	CEMP	3.0%	0.0%	0.0%	23.4%	0.0%	3.1%	50.0%	17.6%	2.0%	0.0%	13.0%	4.3%	5.2%	0.0%
	RMR vs. Unvaccinated		0.0%	0.0%		0.0%	13.4%		35.3%	4.0%	0.0%		33.5%	39.8%	0.0%
**Total 60+**	Covid deaths	15	1	2	37	8	16	79	8	47	1	81	9	29	23
	CEMP	3.8%	0.7%	0.5%	11.9%	10.8%	2.6%	27.0%	10.0%	7.6%	1.1%	17.9%	9.7%	5.9%	2.3%
	RMR vs. Unvaccinated		19.3%	12.3%		91.2%	22.2%		37.1%	28.0%	4.2%		54.1%	33.0%	12.6%
**Total**	**Covid deaths**	**17**	**1**	**2**	**48**	**8**	**17**	**102**	**11**	**48**	**1**	**91**	**10**	**32**	**23**
	**CEMP**	**3.6%**	**0.6%**	**0.4%**	**13.4%**	**8.4%**	**2.7%**	**30.1%**	**11.3%**	**7.1%**	**1.1%**	**17.2%**	**8.6%**	**5.8%**	**2.2%**
	**RMR (versus unvax)**		**17.8%**	**12.2%**		**63.0%**	**19.9%**		**37.7%**	**23.7%**	**3.7%**		**50.2%**	**33.9%**	**12.5%**
	**RMR (versus 1 dose)**			**68.3%**			**31.6%**			**63.0%**	**9.7%**			**67.5%**	**25.0%**
	**RMR (versus 2 doses)**										**15.4%**				**37.0%**

Panel B. Non-White (Black, Hispanic, and Other)

Age		April-Jun 2021 (Alpha)			Jul-Sep 2021 (Delta no Booster)			Oct-Dec 2021 (Delta, With Booster)				Jan-Jun 2022 (Omicron)			
Group	Measure	**0 doses**	**1 dose**	**2 doses**	**0 doses**	**1 dose**	**2 doses**	**0 doses**	**1 dose**	**2 doses**	**3 doses**	**0 doses**	**1 dose**	**2 doses**	**3 doses**
18-39	Covid deaths	1	0	0	6	0	0	14	0	0	0	2	0	1	0
	Other natural deaths	20	4	0	21	4	6	24	3	5	0	22	4	13	3
	CEMP	5.0%	0.0%	NA	28.6%	0.0%	0.0%	58.3%	0.0%	0.0%	NA	9.1%	0.0%	7.7%	0.0%
	RMR vs. Unvaccinated		0.0%	NA		0%	0%		0%	0%	NA		0%	85%	0%
40-59	Covid deaths	6	3	0	21	5	0	36	4	1	0	15	1	3	0
	Other natural deaths	91	25	12	77	14	24	58	26	51	1	110	36	56	37
	CEMP	6.6%	12.0%	0.0%	27.3%	35.7%	0.0%	62.1%	15.4%	2.0%	0.0%	13.6%	2.8%	5.4%	0.0%
	RMR vs. Unvaccinated		182.0%	0.0%		131.0%	0.0%		24.8%	3.2%	0.0%		20.4%	39.3%	0.0%
60-79	Covid deaths	15	3	1	28	2	7	51	3	11	1	40	6	14	4
	Other natural deaths	166	43	60	119	36	108	122	39	131	12	174	38	173	158
	CEMP	9.0%	7.0%	1.7%	23.5%	5.6%	6.5%	41.8%	7.7%	8.4%	8.3%	23.0%	15.8%	8.1%	2.5%
	RMR vs. Unvaccinated		77.2%	18.4%		23.6%	27.5%		18.4%	20.1%	19.9%		68.7%	35.2%	11.0%
80+	Covid deaths	2	0	0	10	3	2	14	1	9	1	25	0	9	1
	Other natural deaths	74	20	44	55	25	62	77	23	74	7	96	18	87	101
	CEMP	2.7%	0.0%	0.0%	18.2%	12.0%	3.2%	18.2%	4.3%	12.2%	14.3%	26.0%	0.0%	10.3%	1.0%
	RMR vs. Unvaccinated		0.0%	0.0%		66.0%	17.7%		23.9%	66.9%	78.6%		0.0%	39.7%	3.8%
**Total**	Covid deaths	7	3	0	27	5	0	50	4	1	0	17	1	4	0
**18-59**	CEMP	6.3%	10.3%	0.0%	27.6%	27.8%	0.0%	61.0%	13.8%	1.8%	0.0%	12.9%	2.5%	5.8%	0.0%
	RMR vs. Unvaccinated		164.0%	0.0%		100.8%	0.0%		22.6%	2.9%	0.0%		19.4%	45.0%	0.0%
**Total**	Covid deaths	17	3	1	38	5	9	65	4	20	2	65	6	23	5
**60+**	CEMP	7.1%	4.8%	1.0%	21.8%	8.2%	5.3%	32.7%	6.5%	9.8%	10.5%	24.1%	10.7%	8.8%	1.9%
	RMR vs. Unvaccinated		67.2%	13.6%		37.5%	24.2%		19.8%	29.9%	32.2%		44.5%	36.7%	8.0%
**Total**	**Covid deaths**	**24**	**6**	**1**	**65**	**10**	**9**	**115**	**8**	**21**	**2**	**82**	**7**	**27**	**5**
	**CEMP**	**6.8%**	**6.5%**	**0.9%**	**23.9%**	**12.7%**	**4.5%**	**40.9%**	**8.8%**	**8.0%**	**10.0%**	**20.4%**	**7.3%**	**8.2%**	**1.7%**
	**RMR (versus unvax)**		**95.4%**	**12.6%**		**53.0%**	**18.8%**		**21.5%**	**19.7%**	**24.4%**		**35.7%**	**40.2%**	**8.2%**
	**RMR (versus 1 dose)**			**13.2%**			**35.6%**			**91.5%**	**113.8%**			**112.5%**	**22.9%**
	**RMR (versus 2 doses)**										**124.3%**				**20.4%**

Table S14. Confidence Intervals for Non-Covid-NMR Estimates in Text Table 1

Table reports 95% confidence intervals for the non-COVID-19 natural mortality risk (NCNMR) ratios reported in text Table 1. For each calendar quarter and age group, we assign the sample population to a vaccine dose category (0, 1, 2, or 3) based on the average number of people in each category (measured at the beginning of each month) over the three months in the quarter. For each of these four populations (within quarter and age group) we measure the number of non-CoVID-19 natural deaths. This provides eight groups: four groups of decedents (by vaccination status) and four groups of non-decedents. We then run logistic regressions (one for each quarter and age group) with an outcome variable equal to 1 for the decedent groups and 0 for the remaining groups. The regressions use as predictors indicators for vaccination status (1 dose, 2 doses, or 3 doses), with unvaccinated as the omitted category. The regressions use frequency weights, which equal the populations of each eight group. Point estimates differ slightly from Table 3, because Table 3 uses mortality counts by month and the logistic regressions use averages for each quarter. In 4Q-2021, there are no non-COVID-19 natural deaths among persons aged 18-59 who received three doses, so the point estimate is zero, with no confidence interval.

		2 doses		3 doses	
	Relative NCNMR	Estimate	95% CI	Estimate	95% CI
Apr-Jun 2021	18-39	5.5%	[0.8%, 40.6%]		
	40-59	32.8%	[22.9%, 47.0%]		
	60-79	32.4%	[27.6%, 38.0%]		
	80+	61.1%	[52.1%, 71.8%]		
Jul-Sep 2021	18-39	34.3%	[16.9%, 69.9%]		
	40-59	33.0%	[24.0%, 45.5%]		
	60-79	31.7%	[27.2%, 37.1%]		
	80+	94.1%	[79.2%, 111.8%]		
Oct-Dec 2021	18-39	36.7%	[18.1%, 74.3%]	0%	NA
	40-59	49.5%	[37.3%, 65.6%]	15.0%	[7.0%, 32.3%]
	60-79	41.9%	[35.7%, 49.1%]	15.1%	[11.6%, 19.6%]
	80+	90.1%	[76.4%, 106.1%]	33.2%	[26.2%, 42.1%]
Jan-Mar 2022	18-39	49.0%	[26.9%, 89.3%]	27.2%	[11.4%, 65.0%]
	40-59	46.9%	[36.9%, 59.6%]	34.8%	[26.9%, 44.9%]
	60-79	68.6%	[59.8%, 78.7%]	35.0%	[30.8%, 39.7%]
	80+	122.5%	[104.3%, 143.7%]	106.7%	[93.1%, 122.1%]

Table S15. RMR Estimates Based on COVID-19 Mortality Rate

Format is similar to text Table 3 but with a different outcome variable. Sample is same as text Table 2. Table shows COVID-19 Mortality Rate (CovidMR) and relative CovidMR versus the unvaccinated, for people vaccinated with 1 dose, 2 doses, or 3 doses. CovidMR is defined as the number of COVID-19 deaths occurring among persons within the indicated age groups with the indicated vaccination status over the indicated period, divided by the estimated population of people in the same age group, vaccination status, and time period. The bottom two sets of rows sum results from the upper rows across broader age groups. Bottom rows covering all ages should be disregarded due to compositional effects; see discussion above.

		**April-Jun 2021 (Alpha)**			**Jul-Sep 2021 (Delta no Booster)**			**Oct-Dec 2021 (Delta, With Booster)**				**Jan-Mar 2022 (Omicron)**			
Age	Measure	Unvax	1 dose	2 doses	Unvax	1 dose	2 doses	Unvax	1 dose	2 doses	3 doses	Unvax	1 dose	2 doses	3 doses
18-39	CovidMR	0.001%	0.000%	0.000%	0.005%	0.000%	0.000%	0.013%	0.000%	0.000%	0.000%	0.004%	0.000%	0.001%	0.000%
	CovidMR ratio to unvax		**0.0%**	**0.0%**		**0.0%**	**0.0%**		**0.0%**	**0.0%**	**0.0%**		**0.0%**	**23.3%**	**0.0%**
40-59	CovidMR	0.007%	0.007%	0.000%	0.039%	0.023%	0.001%	0.087%	0.032%	0.002%	0.000%	0.039%	0.011%	0.007%	0.000%
	CovidMR ratio to unvax		**108.3%**	**0.0%**		**58.9%**	**2.0%**		**37.1%**	**2.0%**	**0.0%**		**27.8%**	**18.0%**	**0.0%**
60-79	CovidMR	0.058%	0.007%	0.004%	0.159%	0.072%	0.010%	0.354%	0.061%	0.037%	0.002%	0.364%	0.093%	0.062%	0.012%
	CovidMR ratio to unvax		**12.0%**	**6.7%**		**45.5%**	**6.6%**		**17.2%**	**10.3%**	**0.5%**		**25.6%**	**17.1%**	**3.2%**
80+	CovidMR	0.054%	0.025%	0.009%	0.275%	0.319%	0.060%	0.556%	0.360%	0.224%	0.040%	0.690%	0.431%	0.366%	0.101%
	CovidMR ratio to unvax		**46.6%**	**16.4%**		**116.3%**	**21.7%**		**64.7%**	**40.2%**	**7.2%**		**62.6%**	**53.1%**	**14.7%**
Total	CovidMR	0.003%	0.003%	0.000%	0.017%	0.011%	0.000%	0.039%	0.014%	0.001%	0.000%	0.016%	0.004%	0.003%	0.000%
18-59	CovidMR ratio to unvax		**115.0%**	**0.0%**		**62.2%**	**2.2%**		**36.2%**	**2.0%**	**0.0%**		**27.0%**	**21.7%**	**0.0%**
Total	CovidMR	0.056%	0.009%	0.005%	0.185%	0.106%	0.019%	0.404%	0.105%	0.067%	0.008%	0.444%	0.147%	0.109%	0.027%
60+	CovidMR ratio to unvax		**15.7%**	**8.3%**		**57.3%**	**10.1%**		**25.9%**	**16.6%**	**2.0%**		**33.1%**	**24.6%**	**6.0%**
All	CovidMR	0.011%	0.005%	0.002%	0.043%	0.030%	0.007%	0.097%	0.031%	0.018%	0.003%	0.086%	0.030%	0.024%	0.012%
	CovidMR ratio to unvaxed		**41.3%**	**20.3%**		**70.1%**	**15.9%**		**32.0%**	**18.9%**	**3.1%**		**35.1%**	**28.3%**	**13.9%**

Table S16. Vaccinee counts by age and month

Table shows the number of one-dose, two-dose and three-dose recipients by age group and month for Milwaukee County over December 2020 through June 2022..

	1 Dose				2 Doses				3 Doses			
	18-39	40-59	60-79	80+	18-39	40-59	60-79	80+	18-39	40-59	60-79	80+
Dec-20	5,439	3,527	1,293	73								
Jan-21	11,418	10,783	15,778	5,018	7,411	5,061	2,008	189			1	
Feb-21	6,551	7,441	35,158	10,890	18,892	16,013	23,395	6,413	12	9	12	2
Mar-21	48,602	50,584	32,704	3,963	32,579	31,695	67,372	18,740	22	18	22	7
Apr-21	37,553	32,220	14,438	1,835	86,433	85,855	100,301	22,120	44	44	36	9
May-21	25,614	22,341	10,514	1,618	114,453	107,811	109,035	22,883	66	69	60	13
Jun-21	22,116	19,326	9,398	1,521	127,107	117,347	112,852	23,310	83	86	71	14
Jul-21	23,101	20,032	9,568	1,540	133,238	121,736	114,704	23,533	106	114	85	17
Aug-21	28,591	23,204	10,381	1,618	139,661	126,162	115,369	23,557	380	711	1,343	197
Sep-21	29,948	23,625	10,433	1,639	148,224	130,847	110,888	22,547	1,703	2,565	7,967	1,437
Oct-21	27,946	21,577	10,036	1,682	147,548	125,199	88,210	18,150	10,420	13,614	32,633	6,051
Nov-21	27,314	19,868	9,398	1,669	137,264	108,684	62,554	11,593	26,265	34,561	60,276	12,873
Dec-21	27,889	18,357	8,663	1,627	116,716	85,192	43,073	7,778	51,876	62,371	81,658	16,917
Jan-22	28,591	17,630	8,221	1,612	106,020	74,360	35,866	6,802	67,262	76,454	90,131	18,000
Feb-22	27,582	16,781	7,844	1,576	104,633	71,620	33,780	6,473	72,318	81,247	92,941	18,406
Mar-22	27,016	16,359	7,691	1,551	103,881	69,956	32,452	6,242	75,033	83,892	94,631	18,682
Apr-22	27,097	16,169	7,411	1,488	103,332	68,749	31,486	6,026	76,648	85,708	96,087	19,016
May-22	27,154	16,020	7,210	1,454	102,818	67,850	30,712	5,816	78,064	87,109	97,240	19,315
Jun-22	27,231	15,894	7,109	1,466	102,431	67,039	30,079	5,613	79,255	88,322	98,143	19,570

Figure S1. Sample selection flowchart

Chart shows the process for selecting the sample of decedents in Milwaukee County used in the vaccine effectiveness analysis. After identifying natural deaths in each time periods, we record natural deaths by vaccination status (0, 1, 2, or 3 doses). Table 2 reports the number of Covid-19 deaths and other natural deaths by period, age bin, and vaccination status.

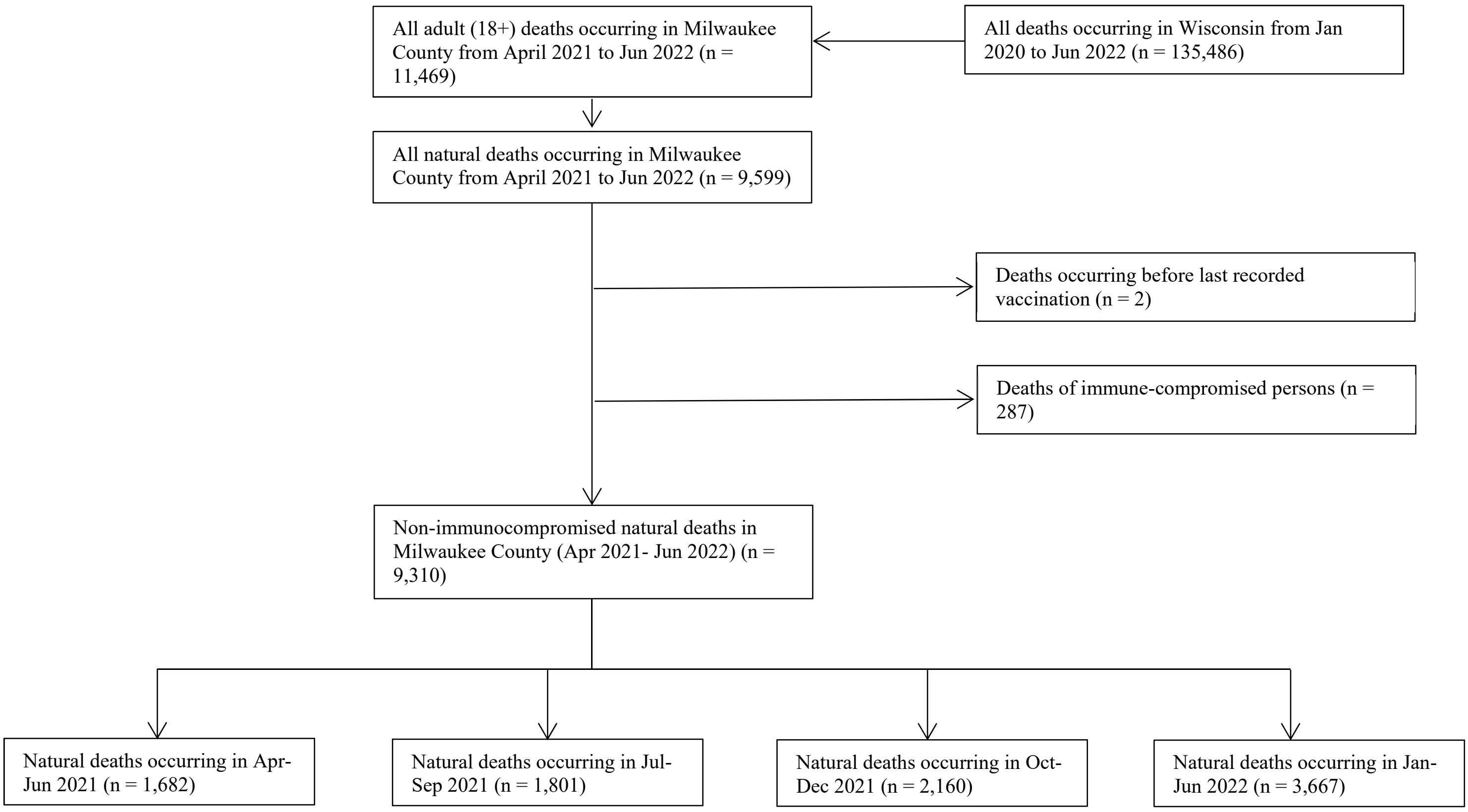

Figure S2. Correlation for Wisconsin and Milwaukee between Natural Mortality in 2019 and COVID-19 Mortality in 2020.

Figure shows scatterplot of natural mortality in Wisconsin (**Panel A**) and Milwaukee County (**Panel B**) over April-December 2019 against COVID-19 mortality over April-December 2020, for groups defined by age (18-39, 40-49, 50-59, 60-69, 70-79, 80-89, 90+)*gender*race/ethnicity, best-fit regression line, and Pearson correlation coefficient. In contrast to text Figure 1, the population groups are not also divided into SES quintiles due to data limitations in Wisconsin during the pre-COVID period.

Panel A. Wisconsin

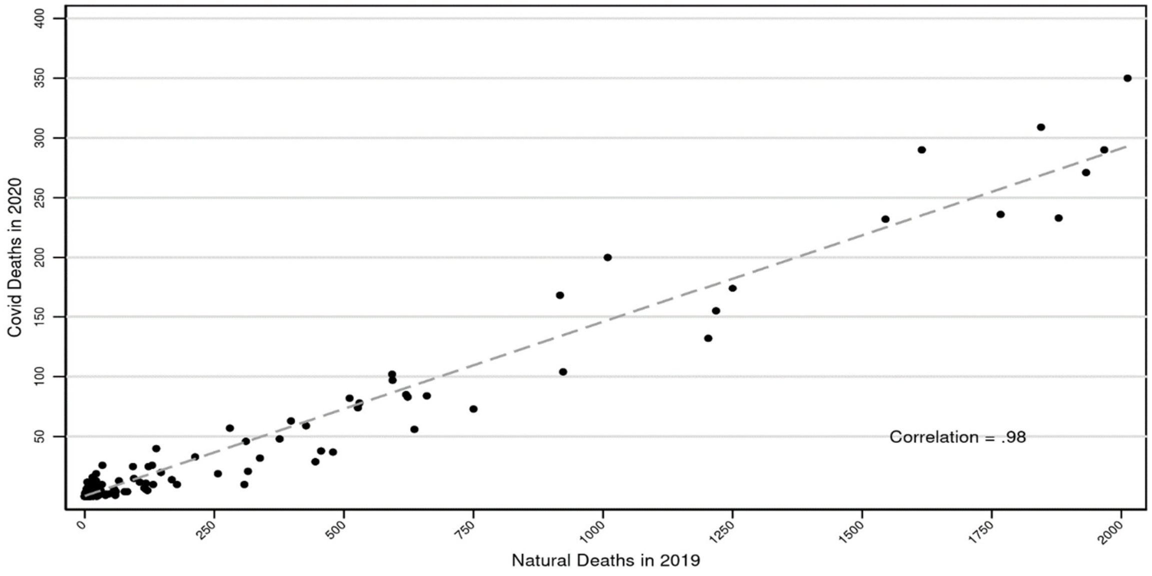

Panel B. Milwaukee County

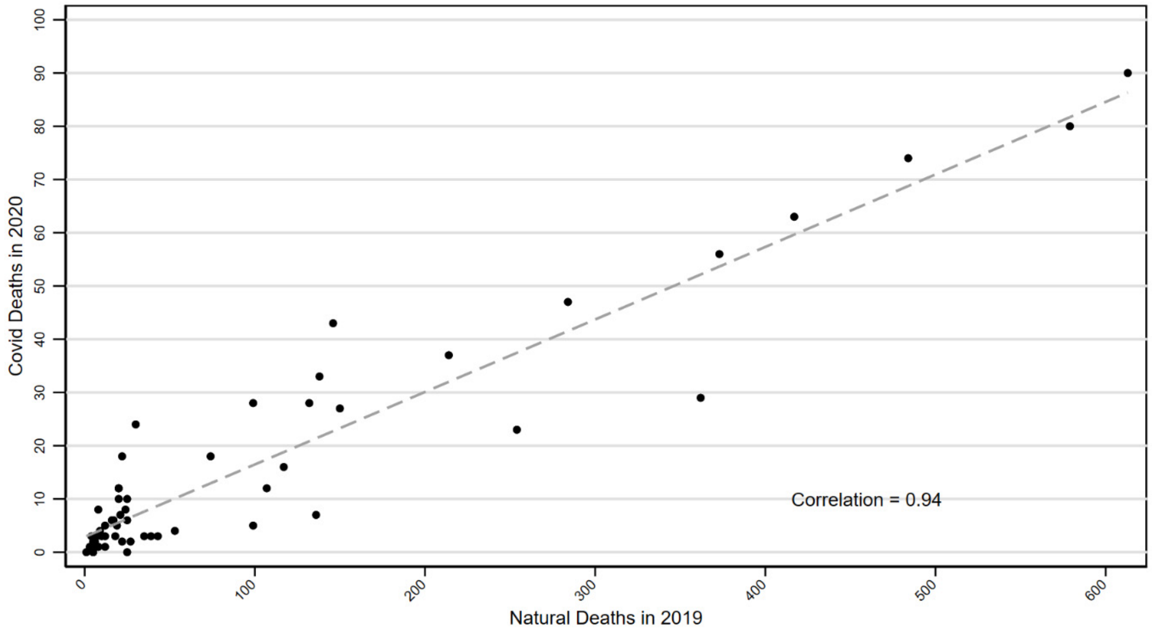

Figure S3. Vaccination Rates for Adults by Age Group in Milwaukee County

Vaccination rates over time for Milwaukee County residents, through June 30, 2022, by age range.

Panel A. Full-vaccination rates

Full vaccination rates. defined as 1 J&J dose, 2 mRNA doses (Pfizer, Moderna, or mixed), or more, as percentage of population.

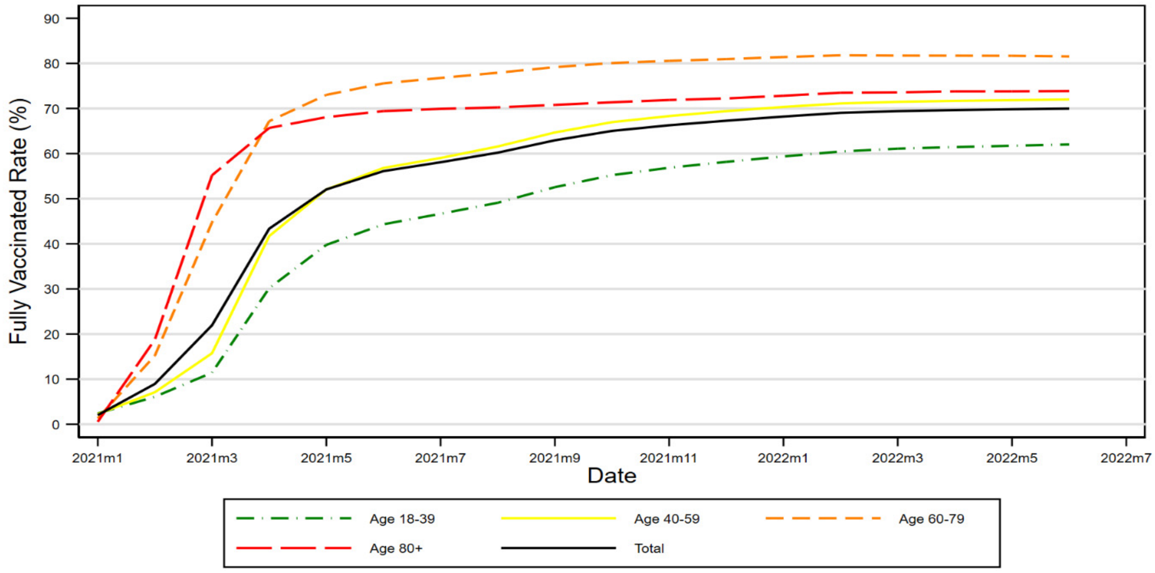

Panel B. Three Dose Vaccination Rates

Three-dose vaccination rates as percentage of people receiving two vaccine doses.

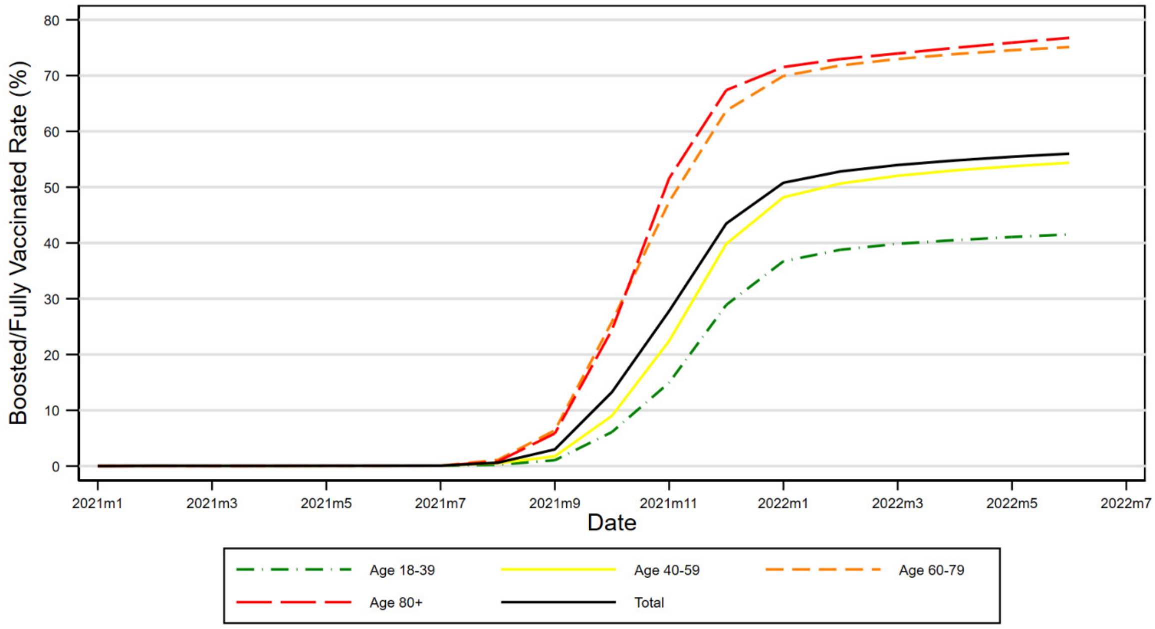

Figure S4. Actual versus Predicted Non-COVID Natural Mortality in Wisconsin During Pandemic Period, with Confidence Intervals

Figure shows monthly data for natural non-COVID-19 deaths for Wisconsin for the pandemic period starting March 2020, predicted natural non-COVID deaths, based on linear extrapolation from 2017-2019 to the same calendar month during the pandemic period. Natural non-COVID-19 deaths (all natural deaths minus COVID-19 deaths) are shown as solid blue line. Predicted natural non-COVID deaths are shown as dashed green line. Dotted lines show upper and lower 95% CIs around predicted deaths. Compared to text Figure 2, the time period is limited to the pandemic period, 95% CIs are added, and the line for total natural deaths is dropped.

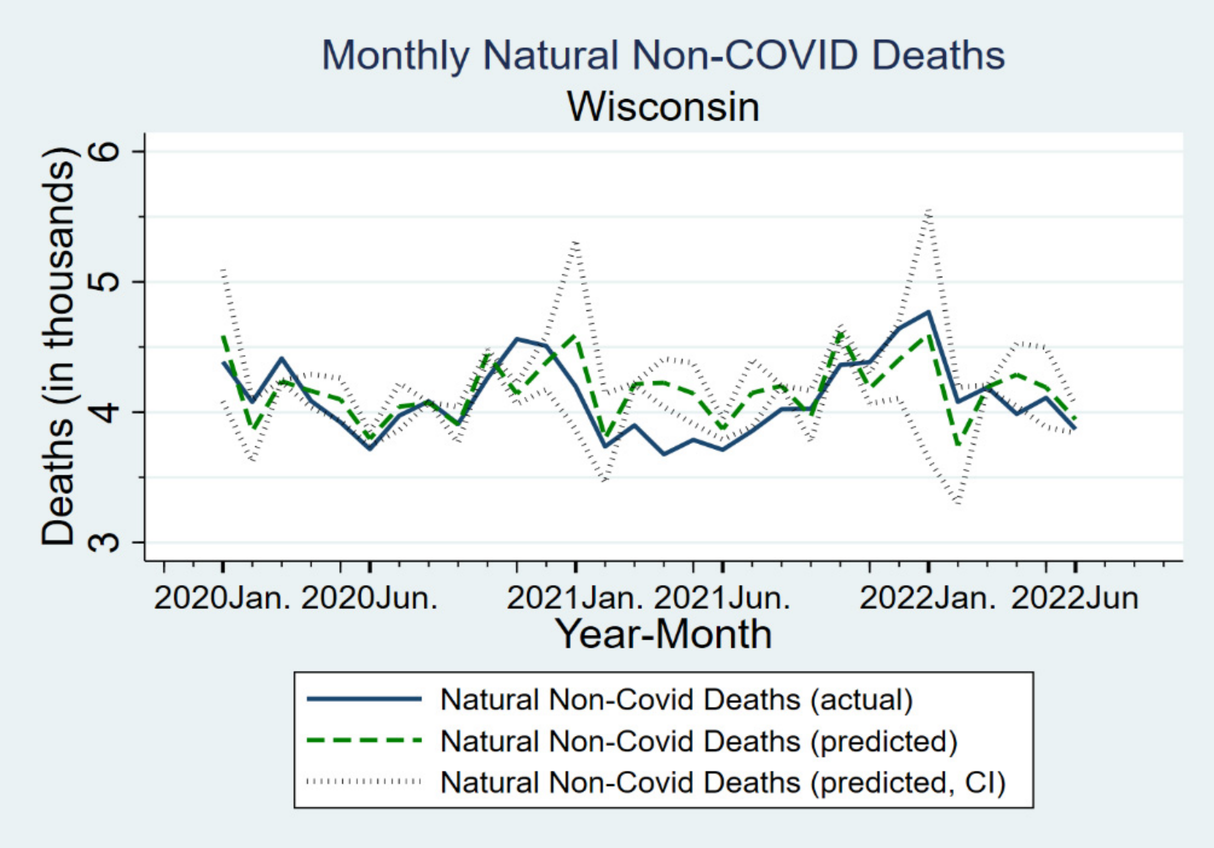

Figure S5. Actual versus Predicted Non-COVID Natural Mortality in Wisconsin During Pandemic Period, By Age Range

Figures are similar to text Figure 2 (left hand graphs) and Figure S4 (right-hand graphs), but are divided into age ranges. **Panel A**. Ages 18-59. **Panel B**. Ages 60-79. **Panel C**. Ages 80+.

Panel A. Ages 18-59.

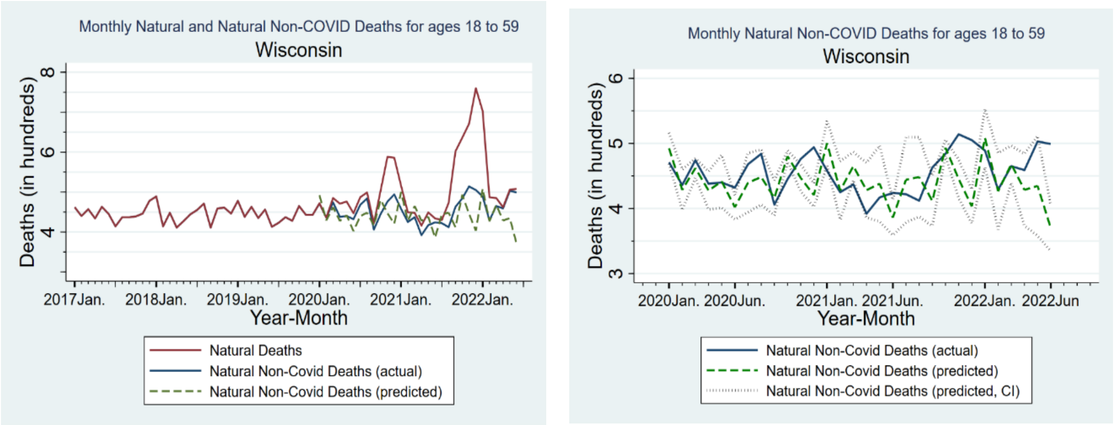

Panel B. Ages 60-79.

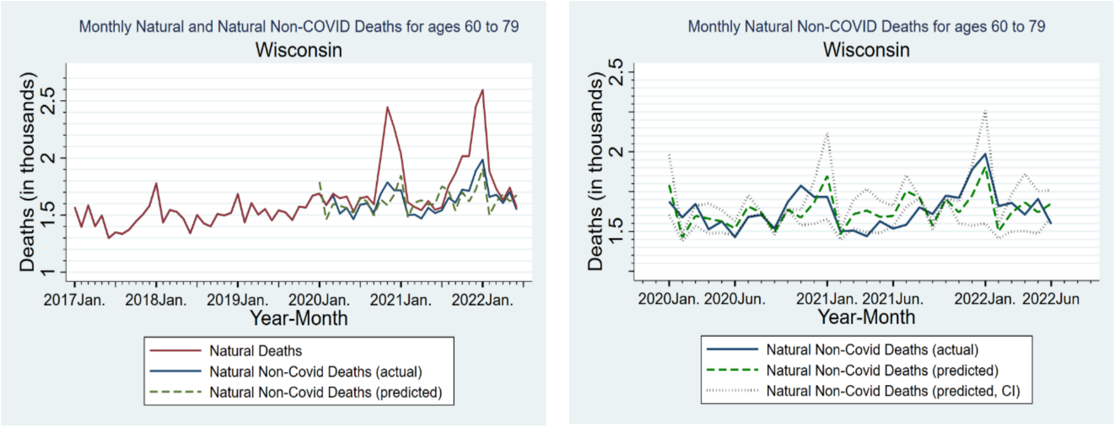

Panel C. Ages 80+

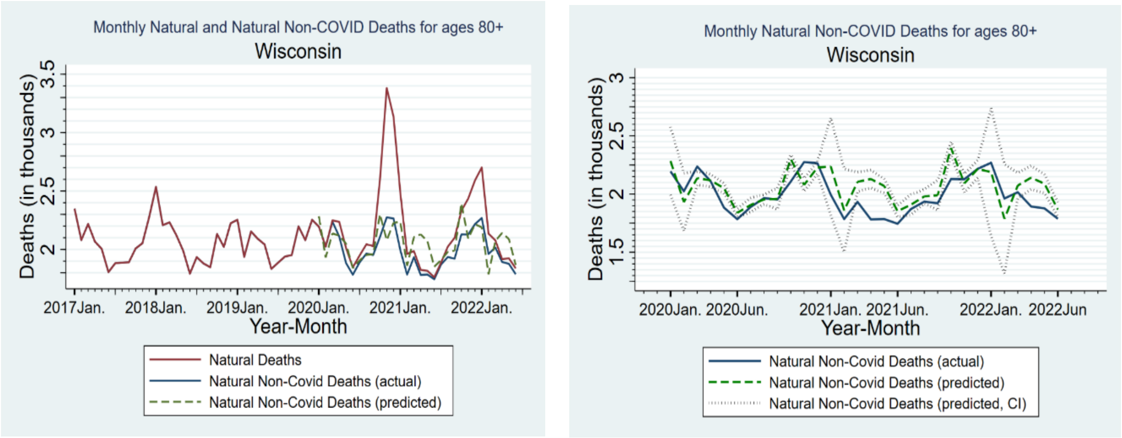

Figure S6. COVID-19 Deaths Counts: Text Fields vs. ICD-10 Codes

Panel A. Blue line (left-hand y-axis) shows monthly COVID deaths data for Wisconsin (Panel A) for 2020—June 2022. Right-hand y-axis (red line) shows difference between COVID-19 deaths, counted using text fields (our preferred approach) and COVID-19 deaths counted using ICD-10 codes from NCHS. **Panel B**. Similar to Panel A, but fur Indiana.

Panel A. Wisconsin

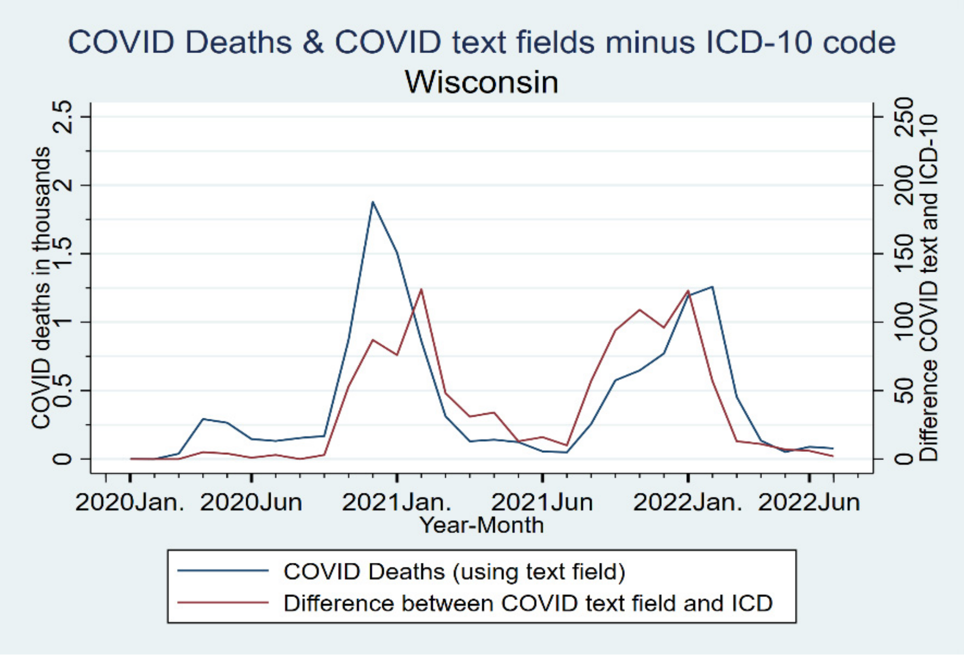

Panel B. Indiana

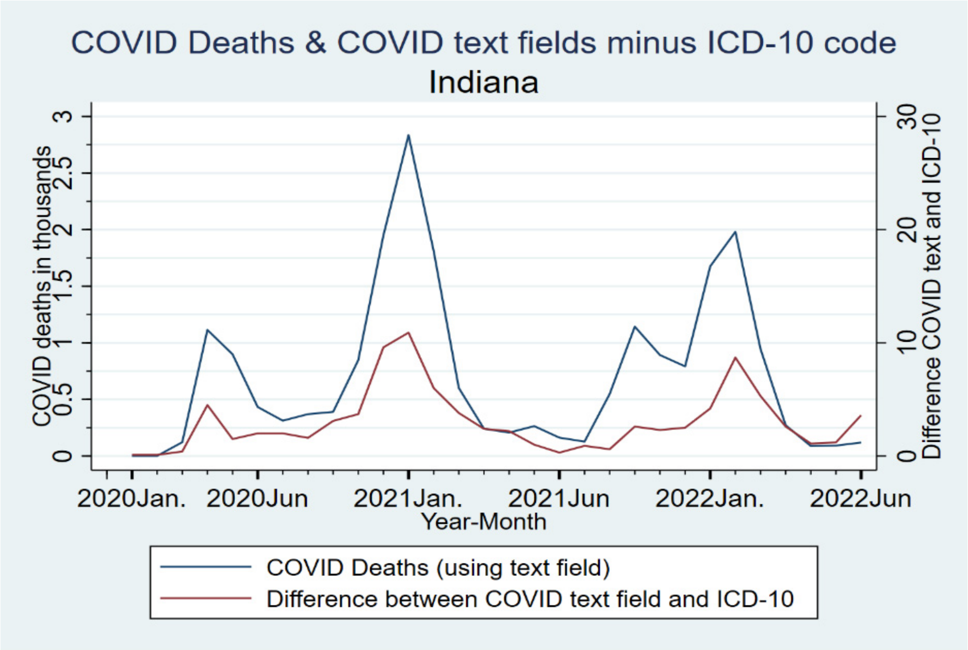

Figure S7. Natural Deaths in 2019 vs. Non-COVID Natural Deaths in 2020

Figure shows scatterplot of natural mortality in Indiana (**Panel A**) and Milwaukee (**Panel B**) over April-December 2019 against Non-Covid natural mortality over April-December 2020, for groups defined by age (18-39, 40-49, 50-59, 60-69, 70-79, 80-89, 90+)*gender*race/ethnicity*zip-SES quintile, best-fit regression line, and Pearson correlation coefficient.

Panel A. Indiana

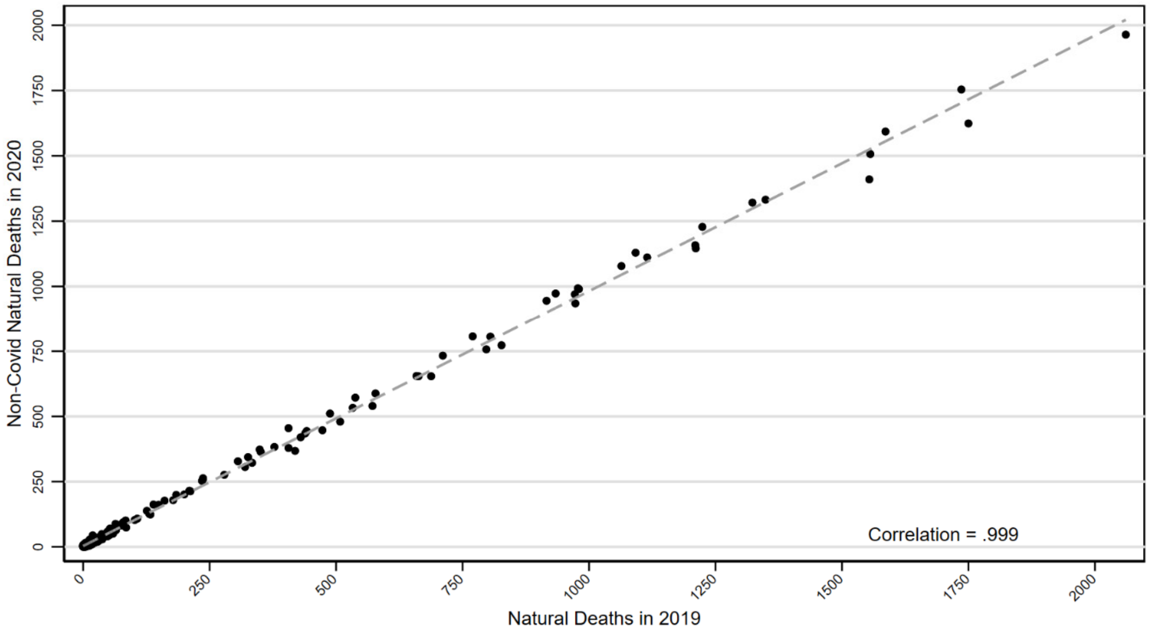

Panel B. Milwaukee County

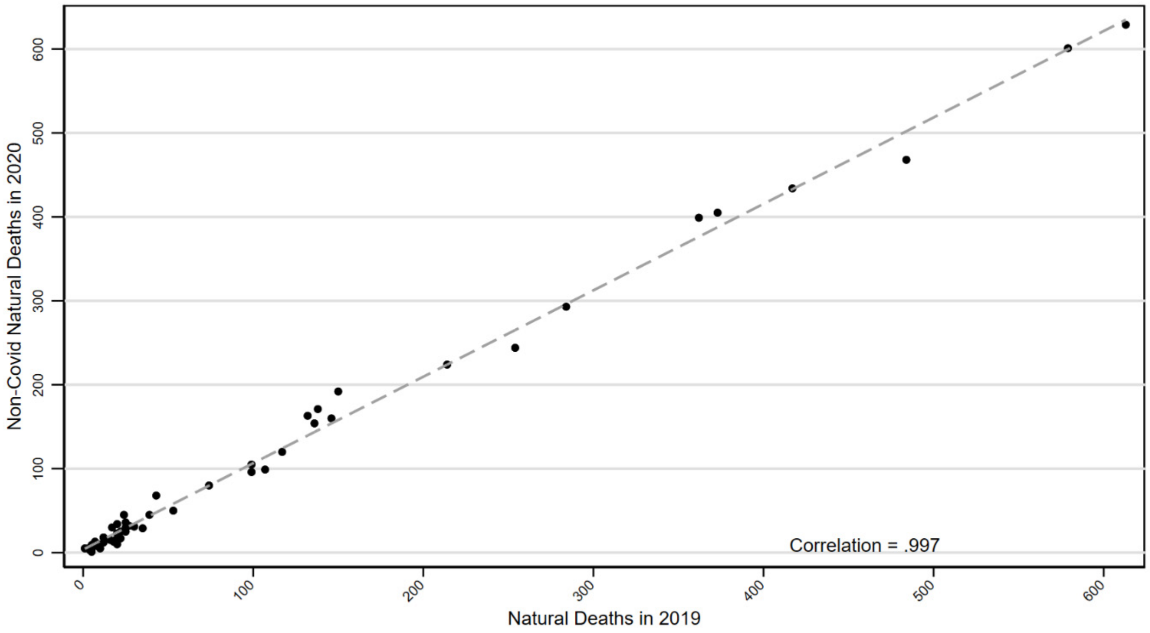

Figure S8. Time-Varying Non-Covid-NMR Rates and Ratios

Figure shows, for ages 80+ (**Panel A**) and ages 60-79 (**Panel B**), monthly rates for total natural mortality (including COVID-19) for the unvaccinated (solid orange line), non-Covid-NMR for the unvaccinated (dashed orange line), and similar data (solid and dashed blue lines) for two- or three-dose vaccinees. Sample is adult decedents in Milwaukee County, Wisconsin, excluding immune-compromised persons and one-dose vaccinees, over April 2021-June 2022. Persons in each group is determined as of the first of each month, lagging vaccination dates by 30 days.

Panel A. Ages 80+

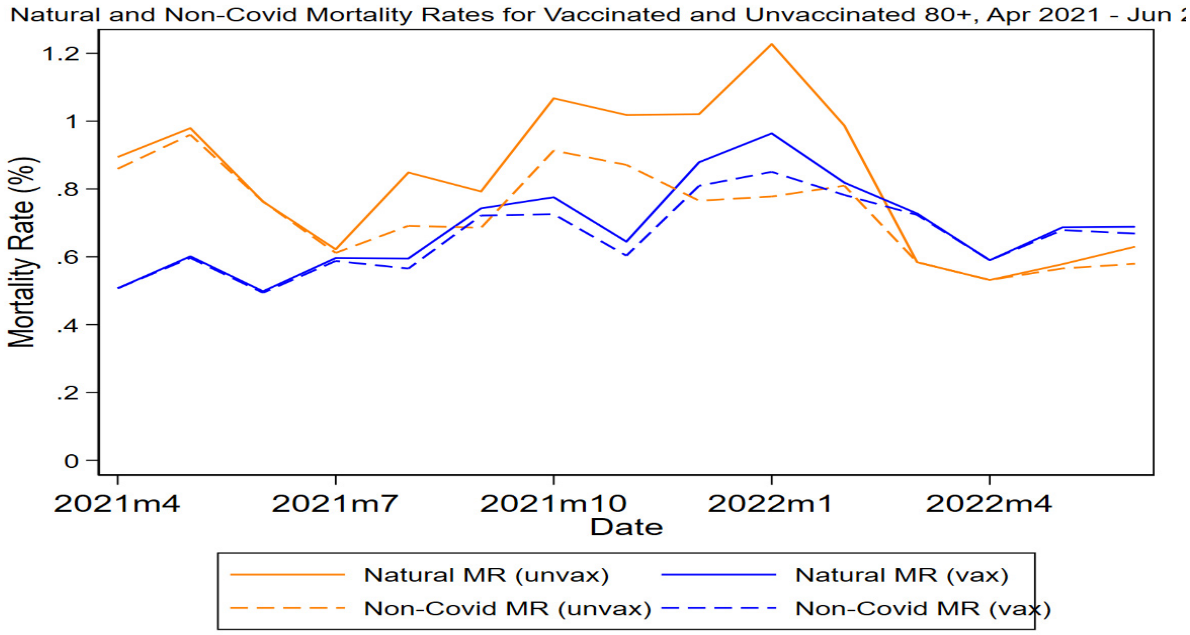

Panel B. Ages 60-79

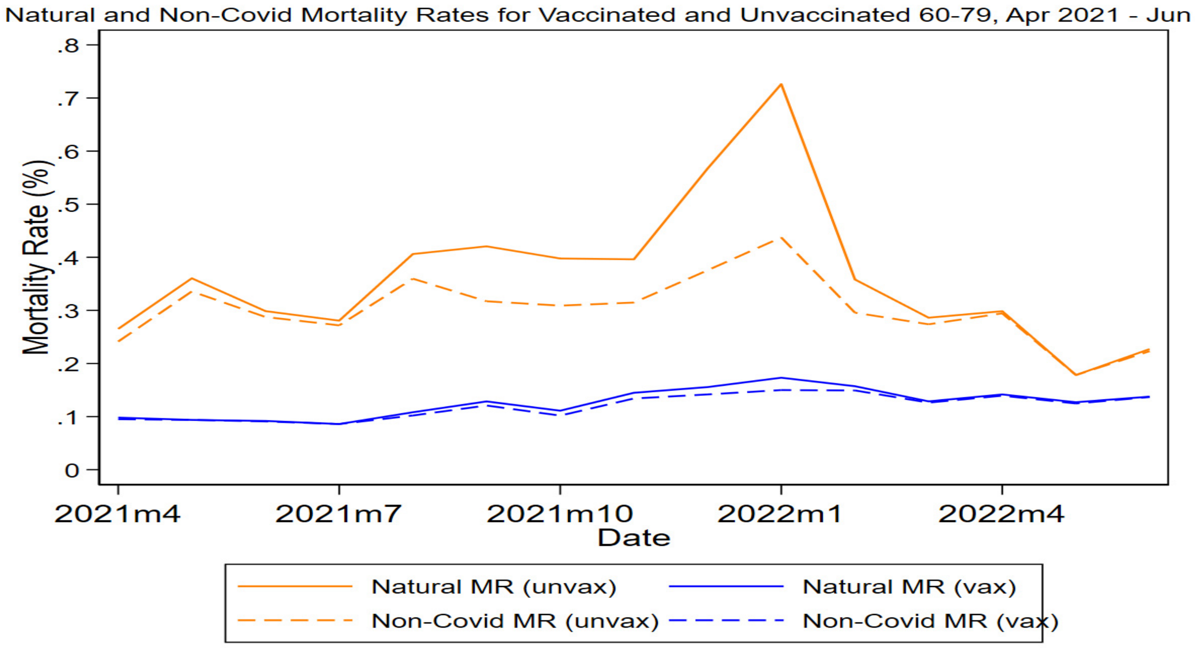

Supplement: Supplementary file 1 [file vaccines-11-00379-s001.zip › vaccines-2152394-SM.pdf]
